# Sizing ocean giants: patterns of intraspecific size variation in marine megafauna

**DOI:** 10.7717/peerj.715

**Published:** 2015-01-13

**Authors:** Craig R. McClain, Meghan A. Balk, Mark C. Benfield, Trevor A. Branch, Catherine Chen, James Cosgrove, Alistair D.M. Dove, Leo Gaskins, Rebecca R. Helm, Frederick G. Hochberg, Frank B. Lee, Andrea Marshall, Steven E. McMurray, Caroline Schanche, Shane N. Stone, Andrew D. Thaler

**Affiliations:** 1 National Evolutionary Synthesis Center, Durham, NC, USA; 2 Department of Biology, Duke University, Durham, NC, USA; 3 Department of Biology, University of New Mexico, Albuquerque, NM, USA; 4 Department of Oceanography and Coastal Sciences, Louisiana State University, Baton Rouge, LA, USA; 5 School of Aquatic & Fishery Sciences, University of Washington, Seattle, WA, USA; 6 Natural History Section, Royal British Columbia Museum, Victoria, BC, Canada; 7 Georgia Aquarium, Atlanta, GA, USA; 8 Department of Ecology and Evolutionary Biology, Brown University, Providence, RI, USA; 9 Department of Invertebrate Zoology, Santa Barbara Museum of Natural History, Santa Barbara, CA, USA; 10 Marine Megafauna Foundation, Truckee, CA, USA; 11 Department of Biology and Marine Biology, University of North Carolina Wilmington, Wilmington, NC, USA; 12 Blackbeard Biologic: Science and Environmental Advisors, Vallejo, CA, USA

**Keywords:** Body size, Megafauna, Allometry, Cline, Intraspecific variation

## Abstract

What are the greatest sizes that the largest marine megafauna obtain? This is a simple question with a difficult and complex answer. Many of the largest-sized species occur in the world’s oceans. For many of these, rarity, remoteness, and quite simply the logistics of measuring these giants has made obtaining accurate size measurements difficult. Inaccurate reports of maximum sizes run rampant through the scientific literature and popular media. Moreover, how intraspecific variation in the body sizes of these animals relates to sex, population structure, the environment, and interactions with humans remains underappreciated. Here, we review and analyze body size for 25 ocean giants ranging across the animal kingdom. For each taxon we document body size for the largest known marine species of several clades. We also analyze intraspecific variation and identify the largest known individuals for each species. Where data allows, we analyze spatial and temporal intraspecific size variation. We also provide allometric scaling equations between different size measurements as resources to other researchers. In some cases, the lack of data prevents us from fully examining these topics and instead we specifically highlight these deficiencies and the barriers that exist for data collection. Overall, we found considerable variability in intraspecific size distributions from strongly left- to strongly right-skewed. We provide several allometric equations that allow for estimation of total lengths and weights from more easily obtained measurements. In several cases, we also quantify considerable geographic variation and decreases in size likely attributed to humans.

## Introduction

*“We tend to pick most ‘notable’ cases out of general pools, often for idiosyncratic reasons that can only distort a proper scientific investigation...Our strong and biased predilection for focusing on extremes (and misconstruing their trends as surrogates for a totality), rather than documenting full ranges of variation, generates all manner of deep and stubborn errors.”*

S.J. Gould (Gould, 1997).

The largest living representatives of most taxa occur in the oceans. Many of these ocean giants have played considerable roles in lore about sea monsters (Carr et al., 2002; Gatschet, 1899; Lenik, 2010; Papadopoulos & Ruscillo, 2002; Paxton, 2009; Verrill, 1897; Woodley, Naish & McCormick, 2011). Today, these formidable species, such as blue whales and giant squids, continue to attract considerable attention from scientists, media, and the public alike. However, misconceptions about the sizes these species obtain are just as rampant in the scientific literature as the popular media.

Quantitative knowledge of body size is vital as it is a significant determinant of an organism’s biological role; and size is the key underlying parameter of many allometric equations that predict a variety of physiological, anatomical, ecological, and life history parameters (Calder, 1984; Peters, 1983). For example, the pervasive pattern relating metabolic rate and body size of mammals, otherwise known as the “mouse-to-elephant” curve, has been observed since the early twentieth century (Benedict, 1938) and continues to inspire discussion of possible causes underlying the relationship (Brown et al., 2004). In mammals, body mass is also positively correlated with the age of first reproduction (Brown et al., 2004), as well as lifespan (Speakman, 2005). Behaviorally, body size in birds has been shown to correlate with flight initiation and distance as an escape mechanism (Blumstein, 2006). Body size also determines the behavior of primates in regard to their habitat usage (Remis, 1995). Among all metazoans, body size is fundamental in structuring trophic relationships (Kerr & Dickie, 2001; Jennings & Mackinson, 2003).

In the absence of detailed observations about the biology of these often rare, elusive, and/or remote marine megafauna, accurate body sizes may provide insights into other aspects of their biology. For example, only 12 complete specimens of the largest invertebrate, the colossal squid, are known. Insights into these organisms are all the more important given that body size may be decreasing due to climate warming (Ohlberger & Fox, 2013) and overfishing (Kuparinen & Merilä, 2007; Genner et al., 2009) and many marine megafauna are listed as vulnerable, threatened, or endangered by the IUCN. Here, we document body size for the largest known marine species of several clades. For these marine megafauna, we also analyzed intraspecific variation and confirmed the largest known individuals for the species. Where data allowed, we analyzed spatial and temporal intraspecific size variation. We also provided allometric scaling equations between different size measurements as resources to other researchers. In some cases, the lack of data prevented us from fully examining these topics and instead we specifically highlighted these data deficiencies and barriers that exist for data collection.

## Methods

Species were chosen that frequently occur in the mainstream media and where sizes were often misreported. Additional taxa were added when data were accessible. A thorough search of the available literature was conducted to find size measurements for the species covered here (Table 1). This included finding data through literature searches via Google Scholar and Web of Science, fisheries data and governmental reports, stranding data, museum records and specimens, online auctions and sales, and personal communications with scientists conducting research on the organisms examined here. All data for each species are available online at http://dx.doi.org/10.5061/dryad.411mv. Analyses were all conducted in R (R Core Team, 2014) including D’Agostino tests for skewness, *t*-tests for differences in mean sizes between groups, and Kolmogorov–Smirnov tests for similarity in distributions, e.g., ocean differences, on intraspecific size distributions. General linear models were also fit to reveal allometric scaling relationships between different body measurements and geographic patterns over temporal or spatial gradients, e.g., year, depth, latitude. All R-scripts for the recreation of the analyses and figures presented here are available at http://dx.doi.org/10.5061/dryad.411mv.

**Table 1:** List of species included in this study with information on the largest known individuals.

Phylum/ Class/ Order	Species	Common name	Record	Largest known confirmed individual
Porifera Demospongiae Haplosclerida	*Xestospongia muta*	Caribbean Giant Barrel Sponge	Largest poriferan	Base diameter: 2.5 m; Volume: 7.24 m^3^
Cnidaria Scyphozoa Semaeostomeae	*Cyanea capillata*	Lion’s Mane Jellyfish	Longest medusozoa	Tentacle length: 36.6 m (note this estimate may not be accurate, see text for discussion)
Cnidaria Scyphozoa Rhizostomae	*Nemopilema nomurai*	Nomura’s Jellyfish	Heaviest medusozoa	Bell diameter: 2 m; Mass: 200 kg
Arthropoda Malacostraca Isopoda	*Bathynomus giganteus*	Giant Isopod	Largest isopod	Total length: 50 cm
Arthropoda Malacostraca Decapoda	*Macrocheira kaempferi*	Japanese Spider Crab	Largest arthropod legspan	Leg span: 3.7 m; Mass: >13.6 kg
Annelida Polychaeta Canalipalpata	*Riftia pachyptila*	Giant Tube Worm	Largest annelid	Tube length: 3 m; Tube diameter: 5 cm; Wet weight: 650 g
Mollusca Bivalvia Veneroida	*Tridacna gigas*	Giant Clam	Largest bivalve	Shell length: 137 cm; Soft tissue mass: 333 kg
Mollusca Gastropoda Caenogastropoda	*Syrinx aruanus*	Australian Trumpet	Largest extant gastropod	Shell length: 72.2 cm
Mollusca Cephalopoda Octopoda	*Enteroctopus dofleini*	Giant Pacific Octopus	Largest octopod	Radial spread: 9.8 m; Mass: 198.2 kg
Mollusca Cephalopoda Teuthida	*Mesonychoteuthis hamiltoni*	Colossal Squid	Heaviest cephalopod and invertebrate	Total length: 4.2 m; Mantle length 2.5 m; Mass: 495 kg
Mollusca Cephalopoda Teuthida	*Architeuthis dux*	Giant Squid	Longest cephalopod	Total length: 12 m
Chordata Chondrichthyes Orectolobiformes	*Rhincodon typus*	Whale Shark	Largest chondrichthyian	Total length: 18.8 m
Chordata Chondrichthyes Lamniformes	*Cetorhinus maximus*	Basking Shark	Largest temperate selachimorphan, second largest chondrichthyian	Total length: 12.27 m
Chordata Chondrichthyes Lamniformes	*Carcharodon carcharias*	Great White Shark	Largest macropredatory selachimorphan	Total length: 7 m (but see text)
Chordata Chondrichthyes Squaliformes	*Somniosus microcephalus*	Greenland Shark	Largest arctic selachimorphan	Total length: 6.4 m
Chordata Chondrichthyes Hexanchiformes	*Hexanchus griseus*	Bluntnose Sixgill Shark	Largest hexanchoid selachimorphan	Total length: 5.5 m
Chordata Chondrichthyes Myliobatiformes	*Manta birostris*	Giant Ocean Manta Ray	Largest batoidean	Disc width: 7 m (but see text)
Chordata Actinopterygii Lampriformes	*Regalecus glesne*	Oarfish	Longest osteichthyan	Total length: 8 m
Chordata Actinopterygii Tetraodontiformes	*Mola mola *	Ocean Sunfish	Heaviest osteichthyan	Total length: 3.3 m; Total height: 3.2 m; Mass: 2,300 kg
Chordata Reptilia Testudines	*Dermochelys coriacea*	Leatherback Turtle	Largest testudines	Curved carapace length: 2.13 m; Mass: 650 kg
Chordata Mammalia Carnivora	*Mirounga leonina*	Southern Elephant Seal	Largest pinniped and carnivoran	Total length: 6.85 m; Mass: 5,000 kg
Chordata Mammalia Carnivora	*Odobenus rosmarus*	Walrus	Third largest pinniped	Total length: 3.8 m; Mass: 1,883 kg
Chordata Mammalia Cetacea	*Physeter macrocephalus*	Sperm Whale	Largest odontocete	Total length: 24 m
Chordata Mammalia Cetacea	*Balaenoptera musculus*	Blue Whale	Largest mysticete, largest cetacean, largest mammal, largest metazoan	Total length: 33 m

## Results and Discussion

### Largest Poriferan: Caribbean Giant Barrel Sponge, *Xestospongia muta* (Schmidt, 1870)

The largest member of the Phylum Porifera is the giant barrel sponge *Xestospongia muta*. *Xestospongia muta* is a dominant component of coral reefs throughout the Caribbean and has been called the ‘redwood of the reef’ because of its large size and estimated long lifespan (McMurray, Blum & Pawlik, 2008). Due to its large biomass, *X. muta* is an important contributor to coral reef habitat heterogeneity (Büttner, 1996) and populations in the Florida Keys and Bahamas are capable of filtering a water column 30 m deep every 2.3–18.0 days (McMurray, Pawlik & Finelli, 2014). The largest known *X. muta* is arguably an individual that served as a scuba attraction off the island of Curaçao in the 1980s and early 1990s. In an article documenting the mortality of this individual in 1997 due to disease, Nagelkerken, Aerts & Pors (2000) reported that the sponge measured nearly 2.5 m in base diameter. The article includes a photograph with a scuba diver for scale that supports the reported size; even if the diver is a tall 1.8 m, the sponge diameter is still clearly longer than the diver’s height (Nagelkerken, Aerts & Pors, 2000).

The factors, if any, which limit the maximum size attainable by *X. muta* remain unknown. Growth of *X. muta* slows with increasing size, but is indeterminate and the largest individuals in the Caribbean have been estimated to exceed 2000 years of age (McMurray, Blum & Pawlik, 2008). Although sponges are functionally clonal, recent work suggests that pumping rates for the largest *X. muta* are reduced relative to smaller individuals (McMurray, Pawlik & Finelli, 2014), potentially reflecting a physiological size or age limit, as has been found for other sponge species (Reiswig, 1974).

Other species in the phylum Porifera are indeed large but probably do not approach the volume of *X. muta*. The congeners *X. testudinaria* and *X. bergquistia* from the Indo-Pacific may attain comparable sizes, however much less is known about the size of these species (Bell et al., 2014). Similarly, several species of glass sponges (class Hexactinellida) may attain large sizes, but investigation of their sizes is limited by the general restriction of hexactinellids to deep-water habitats. For example, a specimen of *Anoxycalyx joubini* from the Antarctic was reported to measure 2 m in height and 1.5 m in diameter (Dayton et al., 1974). Assuming a cylindrical morphology, the volume of this individual was approximately 3.53 m^3^. It should be noted, however, that such calculations produce overestimates of volume, as they do not account for the volume occupied by the innerempty space of the spongocoel which can exceed 50% of solid volume estimates (McMurray, Blum & Pawlik, 2008). Further, an individual of the deep-water hexactinellid *Aphrocallistes vastus* in Saanich Inlet, British Columbia, measured 3.4 m long by 1.1 m high by 0.5 m wide (Austin et al., 2007). If box volume is assumed, appropriate given the shape of *A. vastus*, a volume estimate of 1.87 m^3^ is derived. Species of the genus *Farrea*, a reef building sponge common on the deep summits of seamounts, may also be another contender for largest sponge, although accurate measurements on their size are lacking (McClain, pers. obs., 2008).

**Figure 1:**
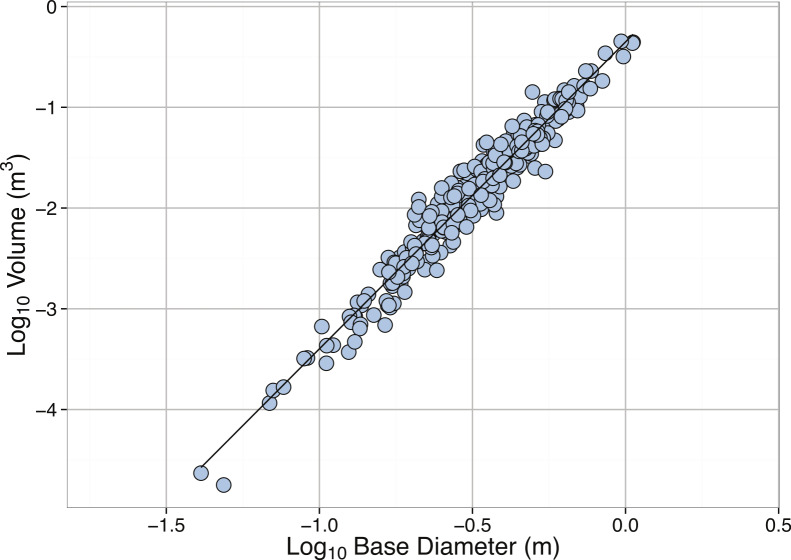
Linear regression between **Log_10_** Base Diameter (m) and **Log_10_** Volume (**m^3^**) for *Xestospongia muta*. See Table 2 for regression equations.

Here we report measurements for base diameter (m), height (m), osculum diameter (m), and volume (m^3^, volume of entire sponge, excluding the spongocoel) largely from the work of McMurray and colleagues on reefs off Key Largo, FL and the Bahamas (McMurray, Blum & Pawlik, 2008; McMurray, Pawlik & Finelli, 2014). Sponge volumes were calculated following the formula in McMurray, Blum & Pawlik (2008). The data gathered here were used to calculate an allometric equation relating base diameter to sponge volume (Table 2, Fig. 1). Using this equation, the 2.5 m specimen from Curaçao is estimated to have a volume of 7.24 m^3^, suggesting that *X. muta* is indeed the largest sponge species.

**Table 2:** Allometric scaling equations for organisms in this study.

Species	Dependent variable	Independent variable	a	s.e.	b	s.e.	*R* ^2^	*p*-value	*N*
*Xestospongia muta*	Volume (m^3^)	Base diameter (m)	−0.35	0.02	3.05	0.04	0.95	< 2.2∗10^−16^	339
*Xestospongia muta*	Base diameter (m)	Height (m)	0.01	0.02	1.02	0.03	0.77	< 2.2∗10^−16^	339
*Xestospongia muta*	Osculum diameter (m)	Base diameter (m)	−0.01	0.01	0.68	0.02	0.81	< 2.2∗10^−16^	339
*Xestospongia muta*	Osculum diameter (m)	Height (m)	0.05	0.02	0.75	0.02	0.75	< 2.2∗10^−16^	341
*Tridacna gigas*	Cost (USD)	Length (cm)	1.30	0.21	2.32	0.12	0.79	< 2.2∗10^−16^	91
*Tridacna gigas* (onlines sales)	Width (cm)	Length (cm)	0.53	0.78	0.64	0.01	0.95	< 2.2∗10^−16^	101
*Tridacna gigas* (wild)	Width (cm)	Length (cm)	4.77	1.91	0.49	0.03	0.87	< 2.2∗10^−16^	39
*Syrinx aruanus*	Cost (USD)	Length (cm)	0.21	0.30	1.09	0.19	0.35	< 2.4*e*∗10^−7^	61
*Enteroctopus dofleini*	Mass (kg)	Interocular eye distance (m)	3.61	0.04	2.64	0.04	0.72	< 2.2∗10^−16^	1712
*Architeuthis dux*	Mass (kg)	Total length (m)	1.40	0.18	0.73	0.21	0.22	0.01	38
*Architeuthis dux*	Mass (kg)	Mantle length (m)	0.67	0.03	0.85	0.11	0.58	1.24∗10^−9^	43
*Architeuthis dux*	Total length (m)	Mantle length (m)	1.59	0.03	1.98	0.15	0.72	< 2.2∗10^−16^	64
*Carcharodon carcharias*	Mass (kg)	Total length (m)	0.99	0.04	3.00	0.06	0.95	< 2∗10^−16^	90
*Hexanchus griseus*	Mass (kg)	Total length (m)	0.67	0.06	3.33	0.17	0.92	< 2.2∗10^−16^	35
*Mola mola*	Mass (kg)	Total length (m)	1.82	0.02	3.19	0.07	0.94	< 2.2∗10^−16^	132
*Mola mola*	Mass (kg)	Dorsal to anal fin length (m)	1.24	0.04	2.55	0.23	0.58	< 2.2∗10^−16^	91
*Mola mola*	Total length (m)	Dorsal to anal fin length (m)	−0.13	0.01	1.03	0.03	0.89	< 2.2∗10^−16^	125
*Dermochelys coriacea*	Mass (kg)	Curved carapace length (m)	2.22	0.12	1.255	0.53	0.22	0.033	16

The largest sponge in the dataset had a volume of 0.7 m^3^ (1.7, 0.97, and 0.82 m in height, base diameter, and osculum diameter, respectively), well below that of the largest reported individual at 7.24 m^3^. The 10 largest individuals occurred on deep portions of the reefs off the Plana Cays and San Salvador, Bahamas. These reefs, particularly the uninhabited Plana Cays, may experience less anthropogenic disturbance (see below) relative to reefs located closer to denser human populations. Additionally, both the Plana Cays and San Salvador are characterized by well-developed reef wall systems, which may offer increased water flow and hence food supply for sponges relative to the more gently sloping reefs found at other study sites. Interestingly, in many locations the sizes of *X. muta* may only reach a volume of less than 1.5 m^3^, e.g., off Key Largo, FL, where the demographics of *X. muta* are best described (McMurray, Blum & Pawlik, 2008; McMurray, Henkel & Pawlik, 2010), the largest individual measured only 0.16 m^3^ in volume. Although many of the largest sponges from this site succumbed to mortality as the result of disease in 2005 (Cowart et al., 2006), including an individual measuring 0.38 m^3^ in volume (S McMurray et al., 2014, unpublished data), *X. muta* typically do not exceed 0.13 m^3^ in volume along the Florida Keys reef tract (Bertin & Callahan, 2008).

**Figure 2:**
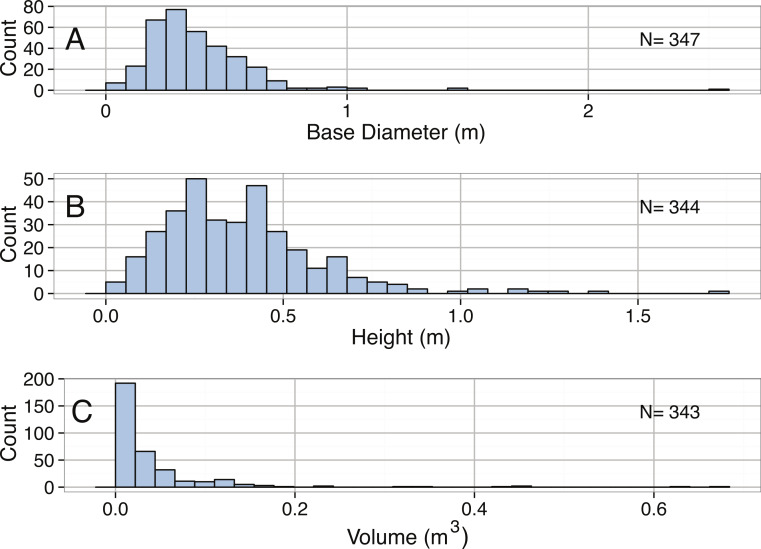
Distribution of (A) Base diameter (m), (B) Height (m), and (C) Volume (**m^3^**) for *Xestospongia muta*.

Distributions of base diameter, height, and sponge volume were all heavily right-skewed and all significantly different from normal distributions (Table 3, Fig. 2). Additionally, *X. muta* in the Caribbean rarely reached diameters over 1 m. Although the survival of *X. muta* has been found to increase with sponge size, stochastic variations in mortality over time due to abiotic and biotic disturbances are likely important in limiting the abundance of the largest individuals (McMurray, Henkel & Pawlik, 2010). Large sponges are particularly susceptible to detachment from the substrata and subsequent mortality from vessel groundings, and the movement of chains, anchors, and derelict fishing gear (McMurray & Pawlik, 2009). The incidence of sponge disease has also increased over recent decades (Webster, 2007) and large *X. muta* have been found to be disproportionately affected by “sponge orange band” syndrome which typically results in sponge mortality (Cowart et al., 2006). A long-term monitoring study of populations of *X. muta* off Key Largo, FL, found that sponge densities have more than doubled as a result of increases in recruitment over the last decade, further contributing to right-skewed size distributions (McMurray, Henkel & Pawlik, 2010; S McMurray et al., 2014, unpublished data). Other monitoring efforts have found similar increases in recruitment throughout the Florida Keys reef tract (R Ruzicka, pers. comm., 2014), although it remains to be seen if these recent patterns are consistent throughout the Caribbean. Finally, it should be noted that all individuals reported here were sampled from depths <30 m due to the limits of SCUBA. Particularly large individuals are often found deeper than 50 m in depth (S McMurray, pers. obs., 2008), however little is known about the size distributions of sponges from mesophotic depths.

**Table 3:** Metrics on intraspecific size distributions. *p*-values are not given for *P. macrocephalus* or *B. musculus* because large sample sizes prevented statistical analysis.

Species	Size metric	Skew	Shape	D’Agostino skewness test *p*-value	75%	90%	95%	Max^a^
*Xestospongia muta*	Base diameter (m)	3.37	Right-skewed	< 2.2∗10^−16^	0.47	0.61	0.69	2.50
*Xestospongia muta*	Height (m)	1.69	Right-skewed	5.14E−10	0.48	0.65	0.76	1.70
*Xestospongia muta*	Volume (*m*^3^)	4.95	Right-skewed	< 2.2∗10^−16^	0.04	0.10	0.14	0.66
*Bathnomus giganteus*	Carapace length (cm)	−0.60	Left-skewed	0.0034	27.00	31.00	33.00	45.00
*Bathnomus giganteus* (mature)	Carapace length (cm)	−0.81	Right-skewed	0.0003	27.50	31.00	33.00	45.00
*Tridacna gigas* (literature)	Length (cm)	−0.04	Normal	0.9214	91.19	102.86	112.90	137.00
*Tridacna gigas* (Pearson & Munro, 1991)	Length (cm)	−1.61	Left-skewed	< 2.2∗10^−16^	86.00	90.00	94.00	106.00
*Tridacna gigas* (online sales)	Length (cm)	0.16	Normal	0.5636	62.55	74.47	86.36	97.79
*Syrinx aruanus* (literature)	Length (cm)	−0.14	Normal	0.8165	57.10	73.04	77.20	91.40
*Syrinx aruanus* (online sales)	Length (cm)	−0.59	Left-skewed	0.0225	53.98	58.42	62.84	72.39
*Enteroctopus dofleini*	Mass (kg)	13.85	Right-skewed	< 2.2∗10^−16^	16.00	18.50	20.50	272.16
*Enteroctopus dofleini*	Interocular eye distance (m)	−1.14	Left-skewed	< 2.2∗10^−16^	0.12	0.13	0.13	0.17
*Architeuthis dux*	Total length (m)	0.67	Normal	0.1147	9.19	12.92	15.26	17.37
*Architeuthis dux*	Mantle length (m)	2.31	Right-skewed	8.28∗10^−8^	1.78	2.36	3.26	7.20
*Architeuthis dux*	Mass (kg)	4.21	Right-skewed	1.21∗10^−8^	163.70	220.00	250.00	907.00
*Cetorhinus maximus*	Total length (m)	0.53	Right-skewed	0.0370	7.10	8.49	8.74	10.00
*Carcharadon carcharias* (literature, mature)	Total length (m)	0.04	Normal	0.7864	4.70	5.37	5.94	8.00
*Carcharadon carcharias* (media)	Total length (m)	0.06	Normal	0.8787	5.38	6.10	6.47	7.62
*Somniosus micorcephalus*	Total length (m)	0.31	Normal	0.2697	3.32	3.96	4.45	6.40
*Hexanchus griseus*	Total length (m)	0.60	Normal	0.1473	2.70	3.01	3.33	5.50
*Hexanchus griseus*	Mass (kg)	0.44	Normal	0.4220	92.50	131.50	145.75	173.00
*Mantra birostris* (global)	Disc width (m)	−0.02	Normal	0.9177	5.25	5.25	5.38	6.20
*Mantra birostris* (Ecuador)	Disc width (m)	0.96	Right-skewed	0.0040	4.86	5.36	5.73	6.20
*Mantra birostris* (Mozambique)	Disc width (m)	−0.83	Left-skewed	0.0048	5.25	5.25	5.25	6.10
*Regalecus glesne*	Total length (m)	0.05	Normal	0.7865	4.00	4.88	5.35	7.72
*Mola mola*	Total length (m)	4.47	Right-skewed	1.06∗10^−11^	1.37	1.81	2.37	3.33
*Mola mola*	Mass (kg)	1.23	Right-skewed	6.25∗10^−5^	162.24	363.60	465.30	2,300.17
*Dermochelys coriacea* (all)	Curved carapace length (m)	−2.92	Left-skewed	< 2.2∗10^−16^	1.60	1.73	1.80	2.13
*Dermochelys coriacea* (mature)	Curved carapace length (m)	0.08	Normal	0.6367	1.64	1.73	1.80	2.13
*Mirounga leonina*	Total length (m)	3.00	Right-skewed	< 2.2∗10^−16^	1.47	1.58	1.75	2.74
*Odobenus rosmarus*	Length (m)	−1.97	Left-skewed	5.11∗10^−15^	3.09	3.25	3.35	3.95
*Odobenus rosmarus*	Mass (kg)	−0.34	Normal	0.0874	1,225.64	1,390.97	1,552.05	1,883.00
*Pyseter macrocephalus*	Total length (m)	0.30			14.33	15.50	15.85	24.00
*Balaenoptera musculus*	Total length (m)	−0.52			25.30	26.52	27.13	33.00

**Notes.**

a Maximum sizes are given from the total dataset but see text and Table 1 for discussion of maximum size in the group as some of the maximum size estimates may not be accurate.

**Figure 3:**
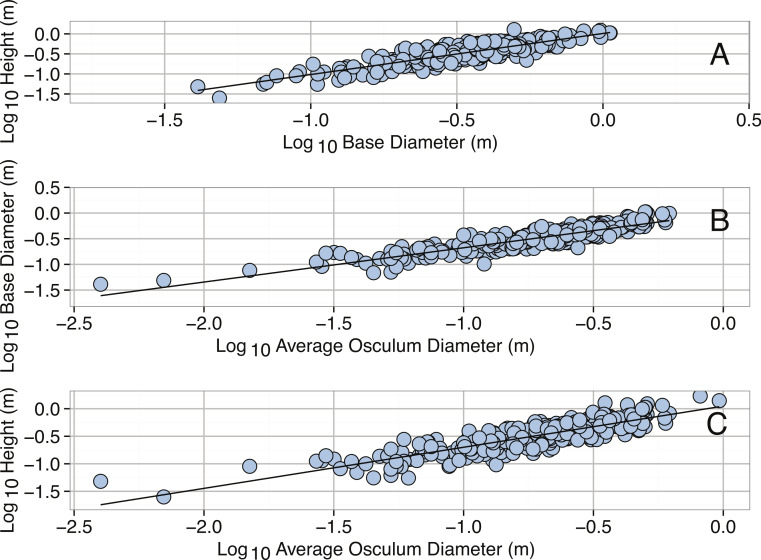
Allometric equations for *Xestospongia muta*. (A) Log_10_ Base Diameter (m) and Log_10_ Height (m). (B) Log_10_ Osculum Diameter (m) and Log_10_ Base Diameter (m). (C) Log_10_ Osculum Diameter (m) and Log_10_ Height (m). See Table 2 for regression equations.

Allometric equations describing the scaling relationships between linear measurements were all highly significant. Base diameter was found to be a significant predictor of height (Table 2, Fig. 3). Similarly, both base diameter and height were found to be significant predictors of osculum diameter (Table 2). These equations are in general agreement with those of McMurray, Blum & Pawlik (2008), who found that the morphology of *X. muta* changes from cone to barrel-shaped with increasing size as osculum diameter widens faster than base diameter.

### Longest Medusozoa: Lion’s Mane Jellyfish, *Cyanea capillata* (Linnaeus, 1758)

Many authorities regard the Lion’s Mane Jellyfish to be the longest of all jellyfish. They are a group of medusavore jellies within the genus *Cyanea*. Many cryptic and described species occur within *Cyanea,* and we therefore report on observations within the genus (Dawson, 2005). On the east coast of the United States, a *Cyanea* medusa was recorded by A Agassiz in an illustrated catalog in 1865 (Agassiz, 1865). He wrote, “I measured myself a specimen at Nahant, the disk of which had attained a diameter of seven and a half feet, the tentacles extending to a length of more than one hundred and twenty feet.” Though this species is reported as *C. capillata*, molecular data from the eastern United States suggests that this species is an undescribed *Cyanea* sp., which is genetically distinct from *C. capillata* in Europe (Dawson, 2005). Nevertheless, this 120-foot (36.6 m) measurement is repeated in both the popular media and scientific literature. No other size measurements of *Cyanea* were found in the literature and we are skeptical of Agassiz’s measurement, as no details are provided on how the measurements were taken.

The ultimate length of *Cyanea* sp. may relate to the fragility of their tentacles and oral arms. In captive scyphozoan jellies, tentacles often tangle with increasing length, and can fragment when knotted together (R Helm, pers. obs., 2014). In wild jellies, tentacles and oral arms may grow substantially longer, but may still break when entangled in marine debris or with other tentacles. Exceptionally long tentacles may also take considerably more time to contract, and thus would be vulnerable to predation. Alternatively, long tentacle trains may increase drag coefficients during ensnaring of large food items.

### Heaviest Medusozoa: Nomura’s jellyfish, *Nemopilema nomurai* (Kishinouye, 1922)

The heaviest Medusozoa is likely *Nemopilema nomurai,* distributed off the coast of China and in the Sea of Japan, where it has received considerable attention in recent years for its massive aggregations. Though only a small number of medusa species have been measured for mass to define the ‘heaviest Medusozoa’, *N. nomurai* is a top contender. *Nemopilema nomurai* can reach ca. 2 m in bell diameter and ca. 200 kg in body mass (Uye, 2008). The medusae are able to gain roughly 2–10% of their body mass per day, depending on size (Uye, 2008). For large 100 kg individuals this would result in growth rates of up to 2 kg per day.

Uye (2008) estimated that an 80 kg *N. nomurai* medusa must consume 14.4 g of carbon per day to meet metabolic and growth demands. Unlike other large pelagic predators like filter-feeding sharks or whales, medusae are unable to actively pursue new food resources if their surrounding seawater becomes depleted. This may place seasonal limits on the upper size of *N. nomurai*. In addition to ecological constraints, interactions between medusa morphology and the surrounding seawater may also limit size. For many medusae, swimming is synonymous with foraging—by moving through the water they create a wake structure that entrains prey (Costello & Colin, 1994). Morphological variation within Medusozoa reflects different modes of swimming and prey capture (Costello, Colin & Dabiri, 2008). For some medusae, such as small jet-swimming hydrozoans, limited scalability of morphology (i.e., muscle tissue) may limit size (Costello, Colin & Dabiri, 2008). In the same way, it is not unreasonable to invoke morphological constraints incurred from larger sizes as setting a size limit on *N. nomurai*.

### Largest Isopod: Giant Isopod, *Bathynomus giganteus* (Milne, 1879)

The giant deep-sea isopod *Bathynomus giganteus* is the largest known isopod species. *Bathynomus giganteus* are abundant scavengers distributed throughout the Gulf of Mexico and Caribbean Sea on upper- and mid-continental slopes at depths typically ranging from 310 to 1800 m, although one individual was recovered from a depth of 80 m (Lemos de Castro, 1978). Though less abundant, giant isopods are also found along the eastern coast of the United States as far north as Georgia (Lowry & Dempsey, 2006). There are records of *B. giganteus* in the Indo-Pacific, however the taxonomy of these samples is under question (Lowry & Dempsey, 2006); thus, we have excluded these records from our dataset and discussion. An individual measuring 76 cm in length was reported in the popular media but cannot be confirmed with actual measurements (Daily Mail Reporter, 2010). The largest giant isopod documented in the scientific literature is a more conservative 50 cm (Lowry & Dempsey, 2006).

**Figure 4:**
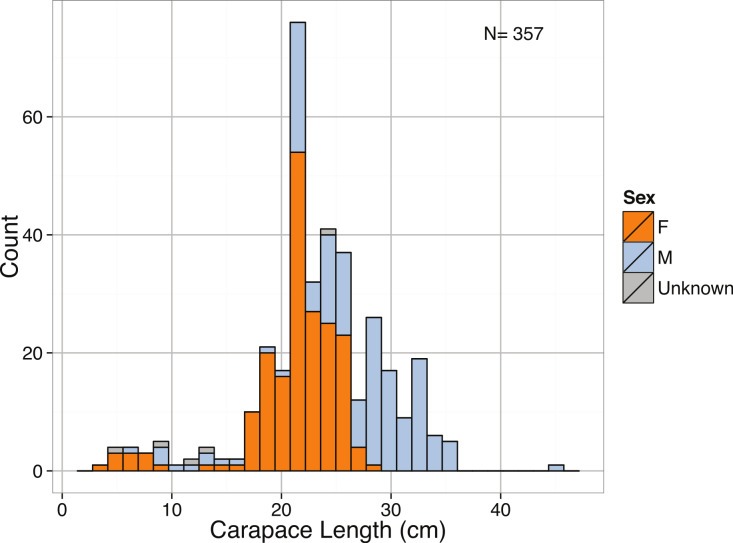
Distribution of Carapace Length (cm) for all individuals separated by sex for *Bathynomus giganteus*.

**Figure 5:**
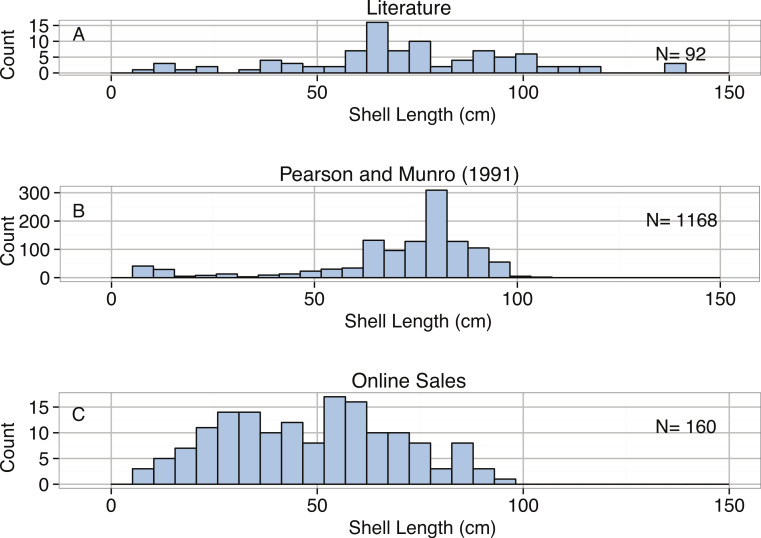
Distribution of Shell Length (cm) from (A) a literature survey, (B) a census by Pearson & Munro (1991), and (C) online sales for *Tridacna gigas*.

Though relatively abundant in the deep sea and common as bycatch in deep trawls, giant isopods are relatively understudied, with few body size measurements available. Holthuis & Mikulka (1972) catalogued all records of *B. giganteus* up through 1972, which forms the major basis of our dataset. As for all of the species here, typical body sizes were found to be much smaller than the largest reported. The distribution of carapace lengths was distinctively left-skewed (Table 3, Fig. 4). When the size distribution was limited to adults (Carapace Length >15 cm), the distribution became right-skewed (Table 3). This tendency toward adults suggests the potential for selection pressure toward larger sizes. We also report here on a previously unknown pattern of sexual dimorphism in size for *B. giganteus* (Fig. 5). We found that adult males were on average 5 cm longer than females (Female mean = 22.1 ± 4.6; Male mean = 27.7 ± 8.8; *p* <2.2e−16).

Timofeev (2001) proposed that deep-sea gigantism, for all crustaceans, is a consequence of larger cell sizes obtained under cold temperatures, as has been proposed for other groups (Van Voorhies, 1996). In crustaceans, deep-sea gigantism may also in part reflect decreases in temperature leading to longer lifespans and thus larger sizes for indeterminate growers (Timofeev, 2001). However, despite little change in temperature beyond the thermocline, deep-sea invertebrates continue to exhibit changes in body size with increasing depth. Alternatively, the maximum potential size of *B. giganteus* may be correlated with oxygen availability, as has been found for related amphipods (Chapelle & Peck, 1999; Chapelle & Peck, 2004). It has been suggested that this relationship arises because the amount of oxygen available controls the amount of sustainable tissue. This hypothesis has been supported experimentally: cell size and cell number both increase with increasing oxygen concentration (Payne et al., 2010). Larger sizes in gastropods are typically found at more oxygenated sites in the deep sea (McClain & Rex, 2001); however, giant isopods are also found in the deep Gulf of Mexico where oxygen concentrations are low.

*Bathynomus giganteus* is a scavenger (Holthuis & Mikulka, 1972; Cocke, 1986; Briones-Fourzán & Lozano-Álvarez, 1991) or facultative predator (Holthuis & Mikulka, 1972; Briones-Fourzán & Lozano-Álvarez, 1991). Specimens in aquaria have survived 8 weeks between feedings (Cocke, 1986) and it is hypothesized that this may be an adaptation for carrying its brood, which would be severely impacted by a full stomach (Briones-Fourzán & Lozano-Álvarez, 1991). However, this may also serve as an adaptation for opportunistic feeding in a habitat with ephemeral food resources. Further support for this hypothesis is the large quantities of lipid reserves in the hepatopancreas (Steves, 1969) and fat bodies (Biesot et al., 1999) of this isopod. Thus, the large size of *B. giganteus* may serve as an adaptation to low and sporadic food availability by increasing fasting potential because greater fat reserves can be maintained (McClain, Boyer & Rosenberg, 2006; McClain et al., 2012b). Larger size also confers a greater foraging area, which is important for both scavengers and predators (McClain, Boyer & Rosenberg, 2006; McClain et al., 2012b).

### Widest Arthropod Legspan: Japanese Spider Crab, *Macrocheira kaempferi* (Temminck, 1836)

*Macrocheira kaempferi* possesses the longest legspan of any arthropod and may be the heaviest extant arthropod. Actual size measurements of *M. kaemperi* are rare, especially in the scientific literature. A classification of recent crustaceans placed the maximum legspan at 4 m (Martin & Davis, 2001). Images of *M*. *kaemperi* are frequent on the internet but often lack measurements. Wikipedia placed the maximum legspan at 3.8 m and the maximum mass at 19 kg. However, none of these measurements can be confirmed. Huang, Yu & Takeda (1990) provided measurements for a considerably smaller mature female off Taiwan, outside of its typical Japanese geographic distribution, that measured 0.242 m in carapace length and 0.184 m wide. A recent specimen on display at the Scheveningen Sea Life center in The Hague, Netherlands has a leg span of 3.7 m and mass greater than 13.6 kg. In terms of mass, the heaviest arthropod is the American Lobster, *Homarus americanus,* with the record holder, according to Guinness World Records, being caught in 1977 off Nova Scotia and weighing 20.14 kg. However, given the lack of mass data for *M. kaempferi* and that specimens of 19 kg are claimed, designating *H. americanus* as the heaviest arthropod may be premature.

It is clear that larger sizes in brachyuran crabs are associated with greater reproductive output in terms of brood weight, number of eggs per brood, and annual fecundity (Hines, 1982). The upper size of marine crustaceans may be limited by oxygen, as noted for amphipods (Chapelle & Peck, 1999; Chapelle & Peck, 2004), but it is unclear whether this is true for arthropods. Dalinger (2011) laid out several hypotheses for the size limits of arthropods living in water that largely center on the size constraints of an exoskeleton. The first is that the time for cuticle regeneration after molt increases with size. For relatively small crabs of 11–14 mm, this can take 8–13 days. Although the cuticle regeneration time for *M. kaempferi* is presently unknown, it may be prohibitively longer at larger masses. Presumably, with a larger cuticle regeneration time, the risk of predation would increase. This longer regeneration time and the time needed between molts may also increase wear and tear on the exoskeleton surface. This damage, although potentially light, may have a cumulative effect that decreases the strength of the cuticle. A larger exoskeleton size also requires disproportionately increasing volumes of molting fluid from the surface area of epidermal cells.

### Largest Annelid: Giant Tube Worm, *Riftia pachyptila* (Jones, 1981)

*Riftia pachyptila* is an iconic deep-sea tube worm found at hydrothermal vents in the East Pacific, and is the largest known annelid (Jones, 1981). It lacks a functional digestive system and derives its nutrition from vent plumes through an endosymbiotic relationship with chemoautotrophic bacteria stored in a specialized organ called a trophosome (Bright & Lallier, 2010). At the hydrothermal vents where they occur, *R. pachyptila* are a dominant source of biomass and act as a foundation species for the vent community (Govenar et al., 2004). *Riftia pachyptila* are considered to be among the fastest growing invertebrates (Bright & Lallier, 2010), and their chitinous tubes can reach up to 3 m in length and 5 cm in diameter at the apex (Grassle, 1986). A large *R. pachyptila* can reach a mass of 650 g wet weight (Fisher et al., 1988). Type specimens were collected at Rose Garden and Garden of Eden hydrothermal vent sites on the Galapagos Rift and 21°N on the East Pacific Rise (Grassle, 1986). The worm itself occupies less than the full length of the tube (roughly less than 2 m for a 3 m tube; Grassle, 1986), concentrated at the apex where its plume can come in contact with hydrothermal effluent. Size distribution studies on *R. pachyptila* are rare, with few studies including more than a few individuals. Those that do provide abundant data are often limited by the uniformity of the sampling regime which selects for homogenous cohorts (Govenar et al., 2004). At this time we are unable to analyze size distributions as data are limited to a community less than one year old and dominated by juveniles (Govenar et al., 2004).

It is interesting to note that the largest annelid is sessile. This releases the species from the biomechanical constraints of movement with a hydrostatic skeleton—a major limitation that may prevent larger sizes (Barnes, 1987). A sessile lifestyle, combined with a ready supply of food derived from chemoautotrophic bacteria utilizing vent fluids, would allow for greater sizes. Indeed, in nematodes the removal of the constraints of limited food supplies and mobility have led to much greater sizes in parasitic over free-living nematodes (Kirchner, Anderson & Ingham, 1980). On the other hand, the ephemeral nature of individual hydrothermal vents (Van Dover, 2000) may prevent the species from reaching larger sizes before mortality occurs.

### Largest Bivalve: Giant Clam, *Tridacna gigas* (Linnaeus, 1758)

As the largest extant representative of the class Bivalvia, *Tridacna gigas* is an important component of Indo-Pacific coral reefs. The body size of *T. gigas* has been extensively researched in the context of aquaculture (e.g., Bell et al., 1997), however size data for wild specimens is scarce. The overall lack of size data for this species is concerning given that *T. gigas* is near-functionally extinct in the wild due to anthropogenic impacts and natural disasters (Neo & Todd, 2013) and is listed as vulnerable by the IUCN. In addition, disturbances to water quality from both natural and anthropogenic sources significantly lower *T. gigas* wet mass and shell length (Elfwing et al., 2003), indicating that size may be an important bioindicator of pollution.

The largest known specimen of *T. gigas*, discovered in 1817 off the northeastern coast of Sumatra, measured 137 cm in length (Knop, 1996). “The mass of the two shells was 230 kg which suggests the live [soft tissue] mass of this animal must have been roughly 250 kg” (Knop, 1996). By mass, a specimen caught in 1956 off the Japanese island of Ishigaki, but not examined before 1984, may hold the record. The shells measured 115 cm in length and the live soft tissue mass weighed 333 kg.

The body size measurements gathered for *T. gigas* included the length of the shell (anterior to posterior), the width (ventral to dorsal, normally described as bivalve height), and height (maximum length perpendicular to the length–width plane). Data were extracted from the literature, museum collections, and personal collections (*N* = 96) and from online sales (*N* = 165). Most of the data, 1,166 measurements, were retrieved from a census taken from Michaelmas Reef in the central Great Barrier Reef, Australia 40 km north-east of Cairns (Pearson & Munro, 1991). Because the original authors of the study could not be contacted, the individual measurements could not be obtained and binned data were extracted.

All body size measurements from the three data subsets (Fig. 5) were less than the 137 cm record holder, and most individuals were much less than 110 cm in length. The size distribution of the population of *T. gigas* at Michaelmas Reef was strongly left-skewed (Table 3). This left-skewed intraspecific distribution also appears to be temporally consistent (Pearson & Munro, 1991). Recruitment of *T. gigas* appears to be low, and recruits settle at 1 to 1.5 cm in length (Braley, 1988). This, combined with a strong decrease in mortality rates as *T. gigas* becomes larger (survival rates are near 100% after shell length of 50 cm; Pearson & Munro, 1991), likely generates this left-skewed distribution. This suggests the potential for predation pressure selecting for larger sizes, further facilitated by a constant food source supplied by symbiotic zooxanthellae. The upper limits of body size for *T. gigas* are most likely constrained by metabolic factors, including food and sunlight availability. In particular, the number of zooxanthellae per unit body mass decreases as clams become larger, potentially restricting the maximum size of *T. gigas* (Griffiths & Klumpp, 1996).

**Figure 6:**
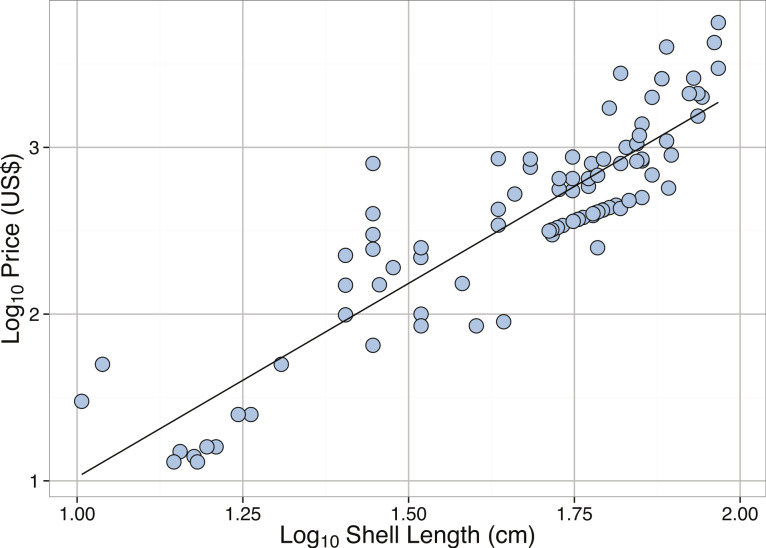
Linear regression between **Log_10_** Shell Length (cm) and **Log_10_** Price (US) for *Tridacna gigas*. See Table 2 for regression equations.

Body size measurements of *T. gigas* from online sales and auctions were compared to field measurements. The 85, 90, and 95% quantiles for the sales data were 71, 77, and 86 cm, respectively. Overall, the sizes of *T. gigas* from online sales were smaller than those found in literature, with median lengths of 70.5 cm in the literature, 78.0 cm at Michaelmas Reef, and 48.9 cm from sales (Fig. 5). The distribution of sizes of *T. gigas* from sales was slightly right-skewed but not significantly different from normal (Table 3). Most of the shells measured from online sales were sourced from commercial clam farms that harvest clams when they reach predefined “adult” sizes. Therefore, given the slow growth rate and long lifespans needed to obtain larger sizes, it may not be economically feasible to allow farmed specimens to reach larger sizes. We do note, however, that increased length of *T. gigas* yields exponentially higher online sales prices (Table 2, Fig. 6).

**Figure 7:**
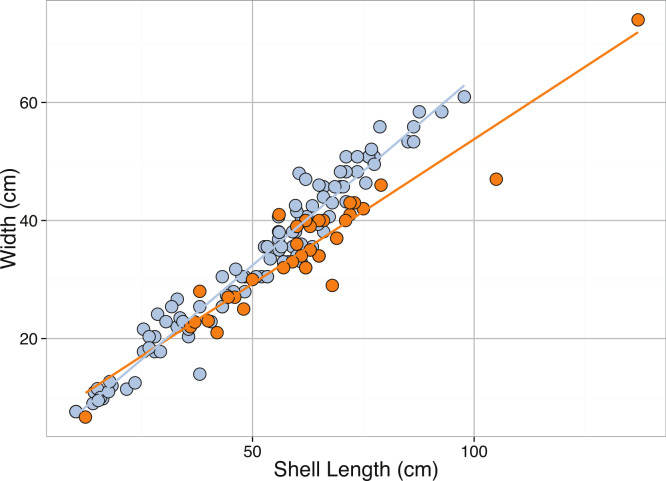
Linear regression between Shell Width (cm) and Shell Length (cm) from online sales and from natural populations for *Tridacna gigas*. See Table 2 for regression equations.

Allometric scaling relationships for shell width and length were calculated, but there were insufficient data for relationships with body mass or shell depth. Scaling relationships to predict width from length for *T. gigas* shells from online sales and wild populations were found (Table 2, Fig. 7). On average, for individuals of the same length, the width of online (i.e., aquaculture) shells were slightly higher compared to wild shells, although these differences were not significant.

**Figure 8:**
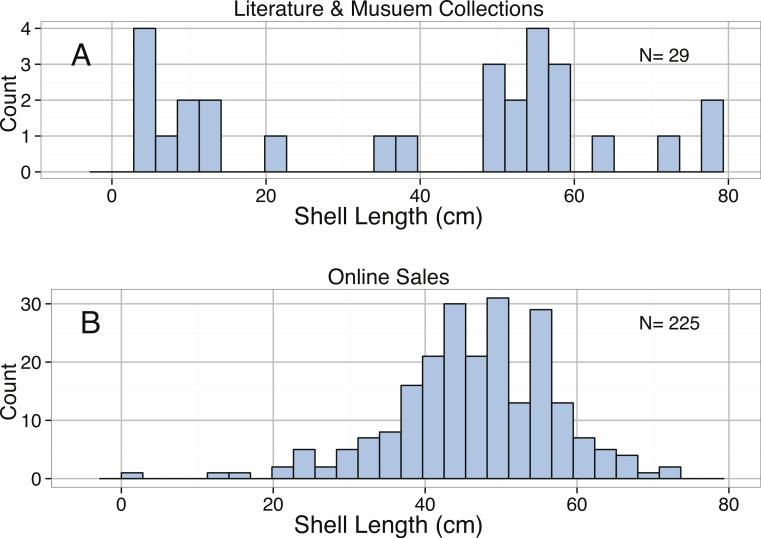
Distributions of Shell Length (cm) from (A) the literature and museum collections and from (B) online sales for *Syrinx aruanus*.

### Largest Gastropod: Australian Trumpet, *Syrinx aruanus* (Linnaeus, 1758)

Distributed from Northern Australia and through the Indonesian Papua New Guinean archipelago, *Syrinx aruanus* is the largest living species of the class Gastropoda. The length of the snail was described by Deshayes as “longeur 3 pouces, 11 lignes” (∼10.6 cm) and the monographer Tryon described the snail to be a modest 20–30 cm in length (Hedley, 1905). Hedley (1905) illustrated the first specimen that measured 58 cm in length and weighed 4.88 kg. Taylor & Glover (2003) reported that largest specimen was 91 cm in length and referenced a 1982 issue of Hawaiian Shell News (Issue 7, pg. 12). A photograph shows club member Don Pisor and children holding the specimen, with the caption stating the specimen was 36 inches (91.4 cm). However, the record holder for the largest *S. aruanus* ascribed by the *Registry of World Record Size Shells* places the maximum length at 72.2 cm. This specimen is also attributed to Don Pisor and was recorded in 1979. We have learned that these specimens are the same individual and the correct measurement is 72.2 cm (D Pisor, pers. obs., 2014); the specimen is currently housed in the Houston Museum of Natural Science. A specimen sold online on 6/8/2011 through eBay UK (http://www.worthpoint.com/worthopedia/syrinx-aruanus-20-0x28-5inc-o13-163769658) was claimed to be 72.4 cm in length. A number of websites claim the existence of a specimen that measured one meter long, but we were unable to confirm this claim.

Limits to the maximum size of gastropods are speculative at this time, but likely reflect energetic constraints. Crawling in gastropods is metabolically expensive compared to almost every other mode of locomotion in the oceans with the exclusion of burrowing in polychaetes (Innes & Houlihan, 1985). Increased surface area of the foot may increase surface friction, adhesion, and drag, thereby reducing efficiency at the largest sizes. Mucus production required for locomotion may also ultimately exceed metabolic scope for such large sizes. In gastropods, up to 80% of ingested energy, but more typically 30%, can be required for mucus production. In an aptly named review, “Mucus from Marine Molluscs,” the authors comment that mucus production in gastropods “is very likely to be more expensive than the respiratory costs of locomotion in many animals” (Davies & Hawkins, 1998). In addition, the physiological costs of calcification may limit the maximum size of shells produced by marine molluscs (Palmer, 1992).

The reported maximum length of *S. aruanus* at 72.2 cm indicates the species is shorter than at least one extinct species. *Campanile giganteum* from the Eocene is the longest fossil gastropod with a maximum reported length of 90 cm (Jung, 1987). Despite having a shorter length, the biovolume of *S. aruanus* is expected to surpass that of *C. giganteum* given the relatively slender shell morphology of the latter species.

**Figure 9:**
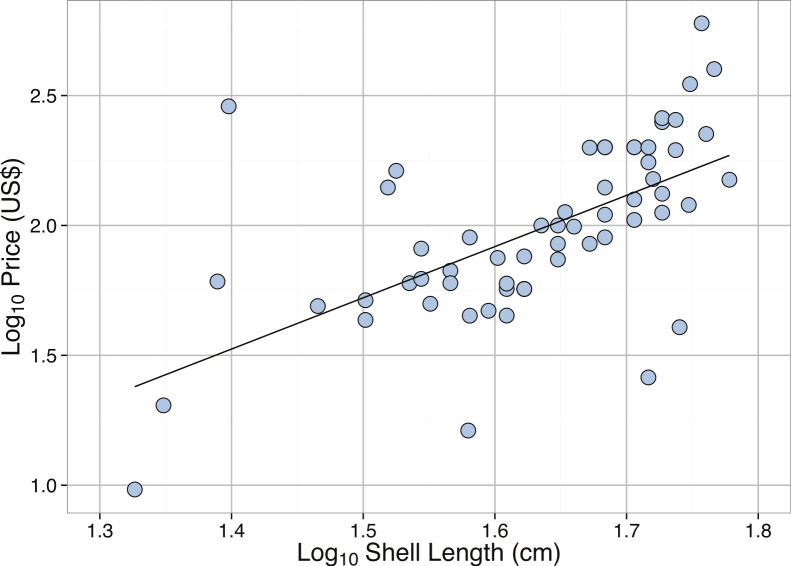
Linear regression between **Log_10_** Shell Length (cm) and **Log_10_** Price (US) for *Syrinx aruanus*. See Table 2 for regression equations.

Body size measurements collected for *S. aruanus* include the length of the shell (maximum length from base to apex along the central axis), the width of the shell (maximum length perpendicular to the central axis), the height of the shell, and the dry mass of the shell. There were minimal size data available for *S. aruanus* in the literature and from museums, with only four specimens listed in Taylor & Glover (2003) and seven specimens at the Delaware Museum of Natural History; therefore, the majority of the data collected were obtained from online auctions. The maximum size for *S. aruanus* found through sales data was 72.4 cm in length (Fig. 8). The distribution of lengths of *S. aruanus* was significantly left-skewed (Table 3). The median length of individuals from the literature and museums dataset was slightly higher (50.8 cm) than that of online sales (45.7 cm); however the mean length of individuals sold online was slightly higher (46.4 versus 41.0 cm) due to a greater representation of the smallest size classes in the literature and museum dataset. Similar to *T. gigas*, larger shells were found to yield exponentially higher online sales prices (Table 2, Fig. 9).

**Figure 10:**
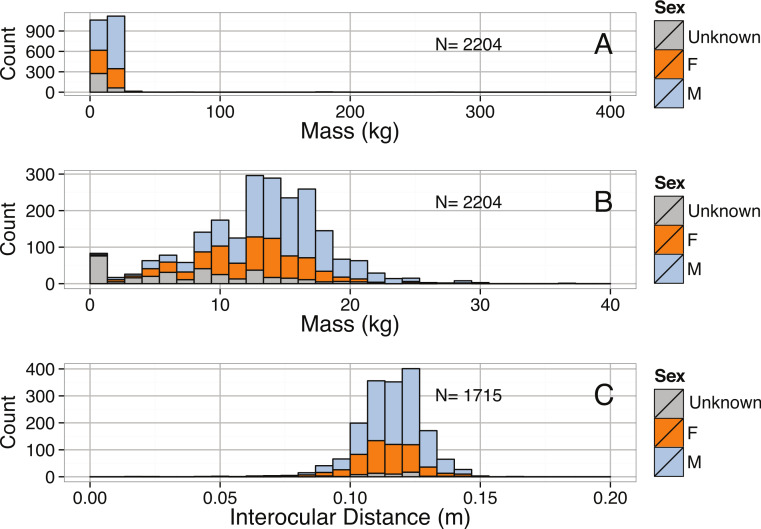
(A) Distribution Mass (kg), (B) distributions of Mass between 0 and 40 kg, and (C) Interocular Distance (m) for male and female *Enteroctopus dofleini*.

### Largest Octopod: Giant Pacific Octopus, *Enteroctopus dofleini* (Wülker, 1910)

*Enteroctopus dofleini*, the largest known species from the class Cephalopoda, is distributed along the coastal regions of the North Pacific, ranging from Korea though Russia and Alaska, and through to California (Nesis, 1987). A review of the largest recorded sizes for *E. dofleini* was provided by Cosgrove & McDaniel (2009). Potentially the largest *E. dofleini*, an individual observed off Port Hardy, British Columbia was reported to weigh 600 pounds (272 kg) with a 32 feet (9.8 m) radial arm span; however, these measurements are estimates, as the specimen was never collected and weighed (Newman, 1994). A specimen caught in the same location in 1956 was collected and weighed; the individual had a radial spread of 8.5 m and mass of 198.2 kg (Newman, 1994). A larger specimen, as measured by radial spread, from Iliuliuk Bay, Unalaska Island in the Aleutians, was reported by Dall (1885) to have a radial spread of 9.8 m. Numerous websites (e.g., Wikipedia) and online fact sheets of the species give the largest specimen as 71 kg, as reported by Cosgrove & McDaniel (2009) when it was brought to the aquarium Undersea Gardens in the 1980s. As noted by Cosgrove & McDaniel (2009), all of the largest *E. dofleini* were caught several decades ago. More recent estimates of maximum sizes are below 57 kg. The contamination of sediments from anthropogenic sources may be impairing *E. dofleini* growth. Anderson (2003) found that individuals typically had high concentrations of heavy metals and PCBs, suggesting that contamination of sediments from anthropogenic sources may be impairing *E. dofleini* growth, and reducing their size at maturity.

Limits to the maximum size of *E. dofleini* may reflect anatomical and energetic constraints related to having a blind gut. The blind gut of *E. dofleini* relegates caloric intake to an installment plan where the entire digestive and excretory processes must occur before additional food is consumed. The inefficiency of this process may ultimately limit energy intake required for growth. This inefficiency may also explain why octopuses on average have the most efficient rates of converting food into body mass. *Enteroctopus dofleini* can grow from a paralarvae of 0.028 g to 18 kg in an average of 34 months (J Cosgrove, pers. obs., 1988). While large size may be an adaptation to reduce predation pressure, it may also constrain the upper size limit. Octopuses rely on hiding and camouflage to reduce predation. Topographically complex habitats, like the rocky subtidal habitat of *E. dofleini*, afford numerous crevasses in which to conceal themselves. Despite the elastic body of octopods, even larger sizes may run out of hiding places. In addition, they have a short life span (no more than 3–5 years) and die after reproducing, thus their maximum size is limited by the speed at which they can grow in a relatively short period of time.

**Figure 11:**
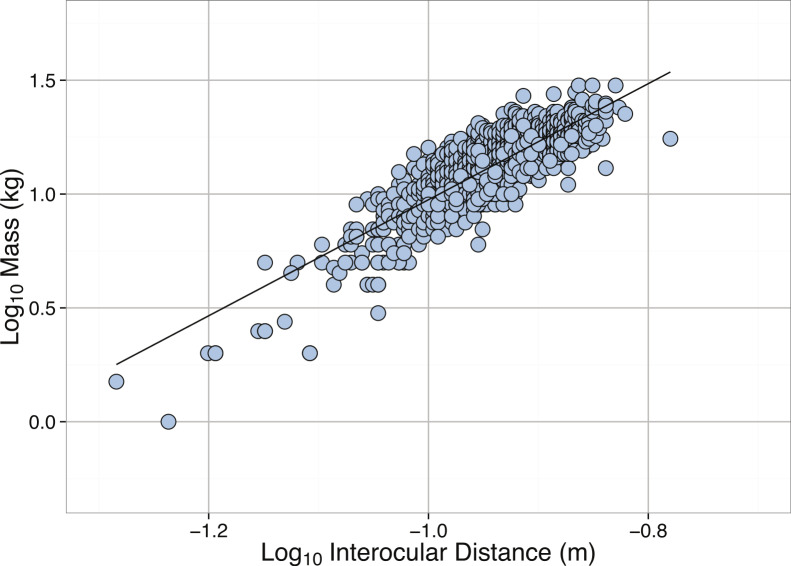
Linear regression between **Log_10_** Interocular Distance (m) and **Log_10_** Mass (kg) for *Enteroctopus dofleini*. See Table 2 for regression equations.

Although data were collected for multiple size metrics, sufficient data exist only for an analysis of body mass and interocular distance of *E. dofleini*. For the limited number of individuals in our data set (Fig. 10), most individuals were well below 30 kg. The distribution of masses was heavily right-skewed and significantly different from normal (Table 3), but interocular distance was left-skewed (Table 3). Males and females differed in both interocular distance and body mass, with males being significantly larger in both cases (*p* = 0.022 and *p* = 0.0352, respectively; Fig. 10). Interocular distance was found to be a significant predictor of body mass (Table 2, Fig. 11).

**Figure 12:**
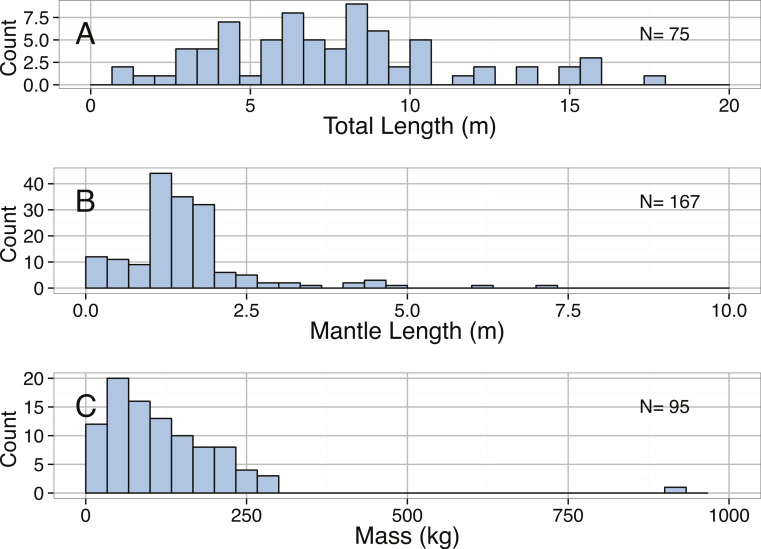
Distribution of (A) Total Length (m), (B) Mantle Length (m), and (A) Mass (kg) for *Architeuthis dux*.

**Figure 13:**
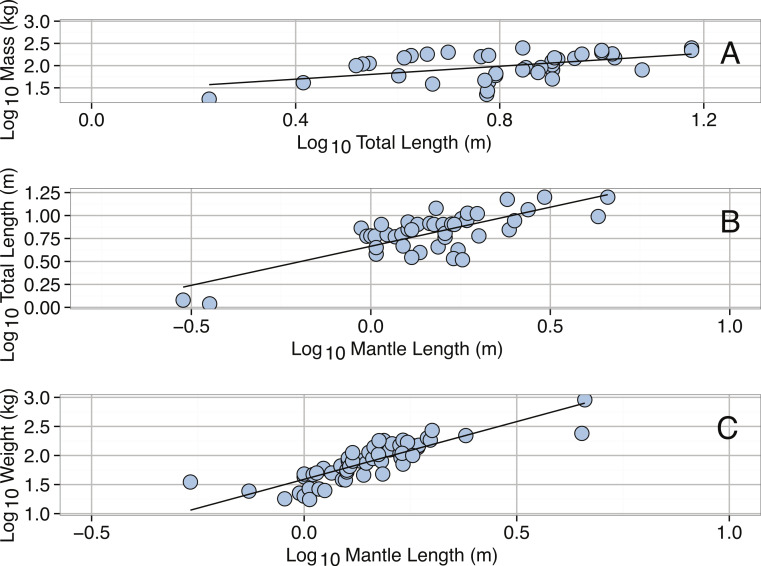
Linear regressions for *Architeuthis dux*. (A) Log_10_ Total Length (m) and Log_10_ Mass (kg). (B) Log_10_ Mantle Length (m) and Log_10_ Total Length (m). (C) Log_10_ Mantle Length (m) and Log_10_ Mass (kg). See Table 2 for regression equations.

### Heaviest Cephalopod and Invertebrate: Colossal Squid,*Mesonychoteuthis hamiltoni* (Robson, 1925)

Of all the marine megafauna listed here, we found the least information for *Mesonychoteuthis hamiltoni*. Only 12 complete specimens are known (another 4 are known from just a fin, mantle, arms, or tentacles) and six of these are juveniles or subadults. The majority of specimens of this species are only known from beaks (*N* = 55). The best preserved and most complete adult specimen was caught on February 22, 2007 by the New Zealand fishing vessel *San Aspiring* while fishing for Antarctic toothfish in the Ross Sea. The total length, including the mantle and tentacles, was 4.2 m with a mantle length of 2.5 m. The total mass was reported as 495 kg. The measurements were confirmed by the Te Papa Museum, where the specimen is currently housed. In 2003, a smaller specimen by mass (300 kg) but with a longer total length of 5.4 m was captured. Although the largest reported giant squid, *Architeuthis dux,* “was estimated to mass 2,000 pounds” (907 kg) by Verrill (1879) based on a Grand Banks specimen from 1871 it is unlikely this is an accurate mass (see discussion below). More typical maximum masses reported in the contemporary literature are 200–280 kg, suggesting that *M. hamiltoni* may reach masses double that of *A. dux*.

### Longest Cephalopod: Giant Squid, *Architeuthis dux* (Verrill, 1879–1880)

The longest cephalopod is *A. dux.* Since the original species description, over 20 species in the genus *Architeteuthis* have been described. However, many of these descriptions are questionable, and new genetic evidence suggests that only a single species exists with minimal genetic variation among ocean basins (Winkelmann et al., 2013). Therefore, herein we treated all size measurements from individuals as *A. dux.*

A substantial amount of size data exists for *A. dux,* including 75 total length measurements, 167 mantle lengths, and 95 mass measurements. The maximum reported length (mantle plus tentacles) of *A. dux* is 17.37 m (Verrill, 1879). The same paper describes three specimens at near 15 m, and several more ranging from 12 m upward and are the largest reported sizes for *A. dux*. A specimen documented in 2002 was reported to be approximately 15 m, but the length was estimated and the actual tentacles were absent. The largest recorded and well-preserved specimen in the contemporary, peer-reviewed literature is 12 m (Bustamante et al., 2008). Given that the few lengths >12 m were not first-hand measurements and come from reported statements, we feel that the longest scientifically verified giant squid is 12 m.

What limits the large size of *A. dux* and *M. hamiltoni* remains unclear. Compared to other molluscs, cephalopods in general have higher metabolic rates (McClain et al., 2012a). The metabolic demands of cephalopods is reflected in the anatomy as coleoid cephalopods consists of two branchial hearts that augment the circulation produced by a main, systemic heart (Barnes, 1987), suggesting selection pressure for increased oxygen delivery. Potentially, metabolic demands would be too great for even larger squids. This may also explain the habitat preference of these larger squids for cold waters (Roper & Boss, 1982) and high mortality rates associated with ocean warming events (Guerra et al., 2011), i.e., the need to slow metabolic rates behaviorally by preferring colder temperature. While also speculative, large sizes may be due to selection for decreased predation pressure as the predators for adult *A. dux* and *M. hamiltoni* are limited to odontocetes.

The intraspecific size distribution of *A. dux* was right-skewed, but not significantly different from a normal distribution (Table 3). The median total length was 7.29 m with 90% of specimens being below 12.9 m in total length (Fig. 12). No significant differences were found between the total lengths of *A. dux* collected in the Atlantic versus the Pacific Ocean, suggesting oceanic variability in size may be minimal (KS Test *D* = 0.0958, *p* = 0.9509). The distributions of mantle lengths and body masses were also heavily right-skewed and significantly different than normal (Table 3). The 900 kg + specimen reported by Verrill (1879) also appears to be an extreme outlier, as the next largest individual only weighed 317.62 kg and 95% of specimens were below 250 kg.

**Figure 14:**
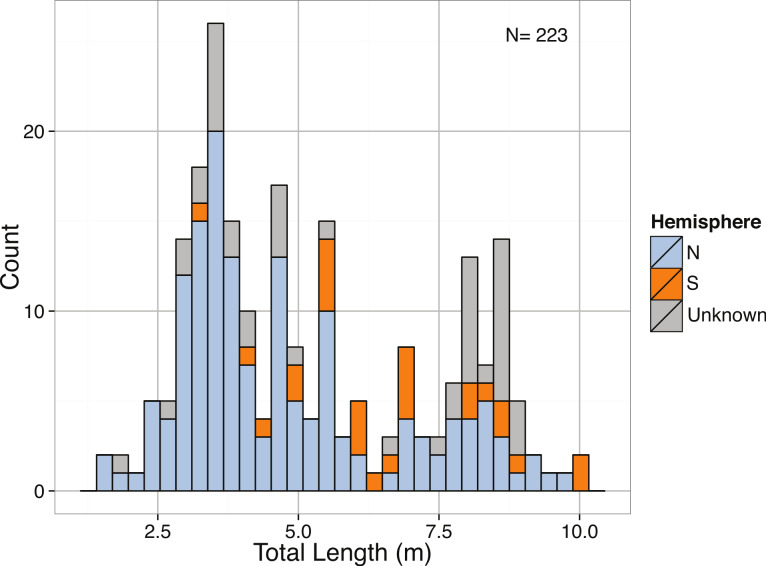
Distribution of Total Length (m) of mature *Cetorhinus maximus* by hemisphere.

We calculated three different allometric equations (Fig. 13). The relationship between total length (TL) and body mass was significant but had low predictive power (Table 2). The relationship between body mass and mantle length (ML) provided better predictive power (Table 2). The improvement in predictive power in the second relationship likely reflects preservation issues resulting in greater changes in tentacle length and that parts of tentacles are often missing; both of which would impact TL measurements. The relationship between mantle length and total length is also significant and provides a useful tool because mantles on specimens are often intact even when tentacles are missing (Table 2).

**Figure 15:**
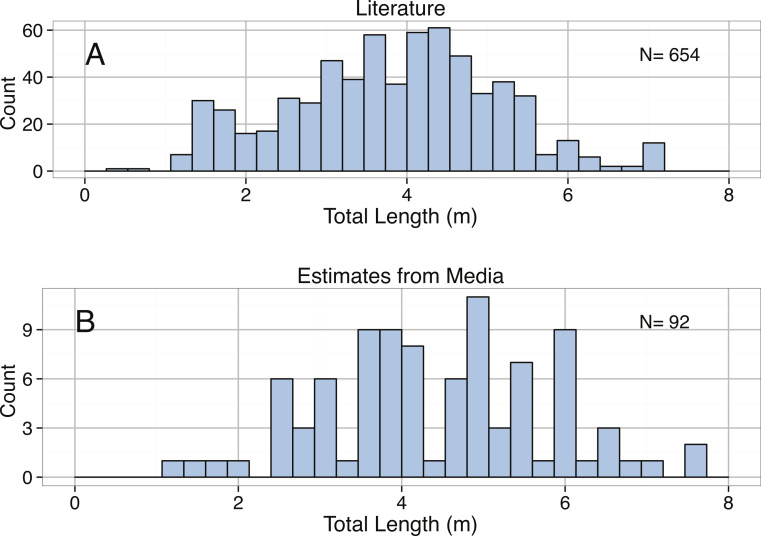
Distribution of Total Length (m) for *Carcharadon carcharias* reported in the (A) literature by sex and (B) media.

### Largest Chondrichthyian: Whale Shark, *Rhincodon typus* (Smith, 1828)

*Rhincodon typus* is a filter-feeding shark distributed globally, and is the largest member of the class Chondrichthyes. Pinpointing an accurate measurement for the largest *R. typus* is difficult. Several informational websites reported the maximum length for this species at 20 m until a blog post published by the lead author questioned the estimates http://deepseanews.com/2013/02/whale-sharks-and-giant-squids-big-or-buhit/. One whale shark FAQ online also states an upper length of 21.4 m is possible (Shark Research Institute, 2014). There is some uncertainty evident in the scientific literature about the largest total length for *R. typus* due to a lack of specifics on how measurements were taken. Chen, Lin & Joung (1997) reported one specimen in the Lotung, Taiwan fish market that measured “approximately” 20 m and weighed 34 tons. The next largest confirmed specimens are all near 18 m. A specimen measuring 18.8 m was reported from the whale shark fishery off the coast of India in the Arabian Sea (Borrell et al., 2011). A reported length of 18 m was given for a female tagged in the Sea of Cortez on July 19, 1996 (Eckert & Stewart, 2001). Another 18 m measurement comes from the Gulf of Thailand in the early 1900s, although the accuracy of the measurement is questioned (Colman, 1997). On April 22, 1975 a female measuring 14.5 m was caught in a drift net off Kollam, India in the Arabian Sea (Devadoss et al., 1990). Based on this evidence, we conclude that the 18.8 m measurement is the most reliable and accurately measured specimen and thus represents the current largest known *R. typus.*

Freedman & Noakes (2002) suggest that selection for planktivorous marine animals favors larger sizes. This reflects both the migration distances required to exploit patches of marine plankton and also the boom and bust nature of plankton blooms: only animals of very large size and energy stores may survive the bust periods. The large size and filter-feeding habits of whale sharks are similar to those of rorqual whales, which are much larger. This does beg the question: “Why don’t whale sharks get bigger?” Whale sharks may be constrained in their upper size by biomechanical limitations of a cartilaginous skeleton, which provides less internal structural support than does a properly calcified bony skeleton like that possessed by baleen whales.

As with the majority of the other species reviewed here, most reported lengths for whale sharks are far below the reported maximum length for this species. Lengths of stranded *R. typus* in South Africa are between 3.4 and 10.26 m (Beckley et al., 1997). An aerial census in the same region estimated no individuals greater than 6–7 m (Cliff et al., 2007). In the Atlantic Ocean near Belize, 317 measured individuals, estimated compared to boat length, had a mean total length of 6.3 m and range of 3–12.7 m (Graham & Roberts, 2007). Individuals aggregating near Holbox Island, Mexico ranged 2.5–9.5 m in length (*n* = 350) (Ramiírez-Maciías et al., 2012). In the Pacific, inshore aggregations in the Gulf of California were primarily comprised of juveniles with total lengths <9 m (Ketchum, Galvaín-Maganña & Klimley, 2013). Another study reported mainly males ranging from 2.5–9 m (*n* = 129) from Bahía de Los Angeles and 2–7 m (*n* = 125) Bahía de La Paz in the Gulf of California (Ramiírez-Maciías, Vaízquez-Haikin & Vaízquez-Juaírez, 2012). At Ningaloo Reef in Western Australia, most of the individuals (*n* = 360) were immature males and between 4–12 m in length with a mean of 7.6 m (Norman & Stevens, 2007). In this study, *R. typus* lengths “were estimated to the nearest 0.5 m total length (TL) using a 15 m rope (knotted at 1 m intervals) held underwater alongside the sharks” (Norman & Stevens, 2007). A subsequent study indicated that the average length, as estimated by a variety of methods, of *R. typus* aggregating at Ningaloo Reef had declined by 2 m from 1995 to 2004 (Bradshaw et al., 2008). An analysis of aggregations of *R. typus* in the Maldives (*n* = 64) ranged from 2.5–10.5 m and averaged 5.98 m (Riley et al., 2010). In the Red Sea, individuals ranged from 3–5 m and averaged 4.3 m (*n* = 87) (Rezzolla & Storai, 2010). As mentioned in these studies, individuals aggregating near coastal regions appear to be numerically dominated by immature males less than 9–10 m in length. Any analysis of whale shark lengths, both maximum and central tendency, is therefore hampered by a startling lack of published information about the biology of adult whale sharks, especially females.

One issue that arises in comparing measurements of *R. typus* is the different methods used to estimate length among studies. Jeffreys et al. (2013) provided an excellent overview of these different measurements.

(1) “Size estimates made to the nearest 0.5 m by experienced water researchers or boat skippers, sometimes estimates are based on the length of a snorkeller or an object of a known size positioned alongside the shark.”
(2) “Measurements made using a tape measure, or a rope knotted at 1 m intervals, held underwater alongside the shark by two swimmers.”
(3) “Size estimations made by driving a boat alongside a shark swimming at the surface and aligning the tip of the tail with the stern of the boat, and estimating total length relative to the bow.”
(4) “Size estimates made by spotter plane pilots by comparison to nearby vessels of a known length.”
(5) “Laser photogrammetry using projected total lengths derived from pre-caudal lengths of free-swimming and deceased shark specimens.”
(6) Some combination of the above.

Allometric scaling equations for *R. typus* focus on predicting total length from other measurements of the individual through the use of laser photogrammetry. The use of pre-caudal length (PCL) to estimate total length (TL) yields a regression of TL (m) = −4.948 + 1.3318 PCL (m) (Jeffreys et al., 2013). Equations have also been provided in other works, e.g., TL (cm) = 20.308 + 1.252 PCL (cm) and TL (cm) = 33.036 + 1.2182 PCL (cm) (Rohner et al., 2011). From Jeffreys et al. (2013) the most robust estimator of TL appears to be *A*_1_ length, the measurement from the leading edge of the spiracle to the bottom of the 5th gill slit (TL (cm) = −38.242 + 5.717*A*_1_ (cm)). The *A*_1_ measurement removes the variability of error associated with swimming undulation on accurately assessing TL. Rohner et al. (2011) found that BP1, the length between the fifth gill and start of the first dorsal fin, yields the best estimates of TL (TL (cm) = 80.994 + 4.8373 BP (cm)).

### Largest Temperate Selachimorphan, Second Largest Chondrichthyian: Basking Shark, *Cetorhinus maximus* (Gunnerus, 1765)

Basking sharks, like whale sharks, are epipelagic sharks, and are the second largest fish in modern oceans (Gore et al., 2008; Skomal, Wood & Caloyianis, 2004; Springer & Gilbert, 1976). *Cetorhinus maximus* primarily inhabit both the Atlantic and Pacific Oceans, but have been found in the Mediterranean, Adriatic, and Indian Oceans (Francis & Duffy, 2002; Natanson et al., 2008). Although typically found near the coasts to the edges of continental shelves, *C. maximus* can travel at depths of 200–1,000 m when migrating (Ebert, Fowler & Compagno, 2013). They feed on abundant planktonic crustaceans in these near-shore environments from the spring through the summer, and spend the winter offshore in deeper environments (Francis & Duffy, 2002; Gore et al., 2008). The majority of studies on *C. maximus* come from these summer environments, as capturing individuals during the winter months when the species is supposedly in deeper water and off the continental shelves is difficult (Valeiras, López & García, 2001), but see Francis & Duffy (2002), Gore et al. (2008), Parker & Boeseman (1954) and Skomal, Wood & Caloyianis (2004). Currently, *C. maximus* is listed as vulnerable by the IUCN.

The largest specimen ever recorded was 12.27 m, and was entangled in a herring net on August 6, 1851 in Musquash Harbor, Bay of Fundy, New Brunswick, Canada (Carwardine, 1995). The limits to maximum size may reflect a combination of biomechanical and energetic constraints. As an obligate ram-filter feeder, *C. maximus* feeds with its mouth open while swimming near the surface (Sims, Fox & Merret, 1997). As a result, drag is increased, as well as energy to capture prey (Sims, Fox & Merret, 1997). It has been suggested that *C. maximus* must forage in areas of dense zooplankton as an optimal foraging strategy (Parker & Boeseman, 1954). *Cetorhinus maximus* is an efficient filter feeder even at lower than expected zooplankton densities (although still dense), and swims at considerably slow speeds while feeding (Sims, 2009; Sims, Fox & Merret, 1997). While *C. maximus* can gain enough energy at its current size, it has tacitly been suggested that an increase in size can (1) increase drag, and thus require more energy to collect enough zooplankton, and (2) limit zooplankton consumption to only dense areas, as lower densities of zooplankton would not be optimal for *C. maximus* to forage (Parker & Boeseman, 1954; Sims, Fox & Merret, 1997).

The total lengths of *C. maximus* in our dataset ranged from 1.53 m (Sims, Fox & Merret, 1997) to 10 m (Hernández et al., 2010) with a mean of 5.2 m. The body size distribution of *C. maximus* was slightly right-skewed, with possible bimodality (Fig. 14), and was significantly different from normal (Table 3). Mean total length of *C. maximus* varies somewhat among ocean basins (Atlantic: 4.67 m, *N* = 216, Indian: 5.5 m, *N* = 70, Mediterranean: 6.66 m, *N* = 73, Pacific: 5.04 m, *N* = 71), and a significant difference in mean total length between hemispheres (North: mean = 4.74 m, South: mean = 6.64 m, *p* = 1.747 × 10^−5^). This could be due to higher concentrations of phytoplankton in the Northern Hemisphere where *C. maximus* are distributed relative to the Southern Hemisphere, as well as the greater availability of coastal habitat in the Northern Hemisphere (Yoder et al., 1993).

**Figure 16:**
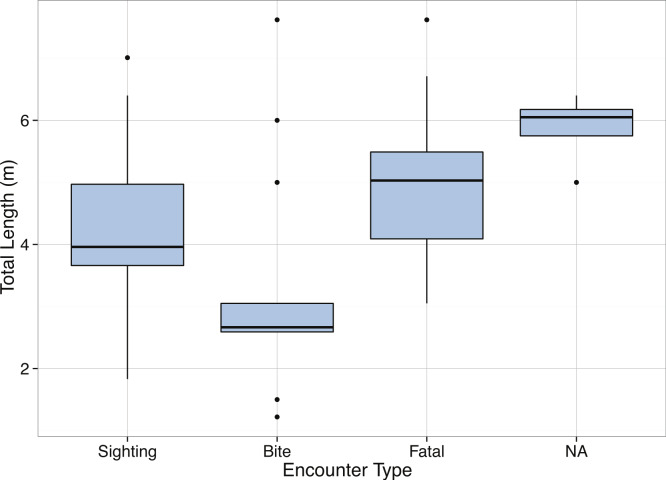
Boxplots of Total Length (m) as reported in the media for *Carcharadon carcharias* by encounter type.

**Figure 17:**
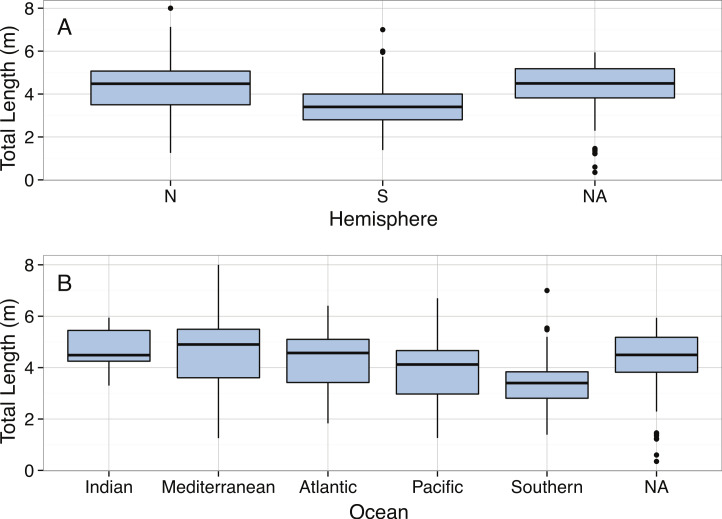
Boxplots of Total Length (m) of mature *Carcharadon carcharias* by (A) Hemisphere, and (B) Ocean.

### Largest Macropredatory Selachimorphan: Great White Shark, *Carcharodon carcharias* (Linnaeus, 1758)

Of the lamnid sharks, the great white shark, *Carcharodon carcharias*, is the one that receives much media attention—both fictional and non-fictional—despite the fact that their ecology is only just starting to be understood (Bruce, Stevens & Malcolm, 2006; Klimley & Ainley, 1998; Weng et al., 2007). *Carcharodon carcharias* inhabits near-shore, coastal environments from temperate to tropical waters (Compagno, 1984), but is known to undergo long migrations to the south-central Pacific (Bonfil et al., 2005; Bruce, Stevens & Malcolm, 2006; Weng et al., 2007).*Carcharodon carcharias* is an apex predator that feeds on large fish and marine mammals (Klimley & Ainley, 1998) and is also known to scavenge on large cetaceans and other carcasses (Long & Jones, 1996).

A large amount of controversy surrounds the length of the largest *C. carcharias.* The two largest reported lengths are over 7 m. An individual, designated KANGA, was caught near Kangaroo Island in Australia on April 1, 1987 and estimated to be over 7 m long. On April 16, 1987 an individual, designated MALTA, was caught off Malta and estimated to 7.13 m long. Although, these estimates drew controversy, and some suggested the specimens were closer to 5–6 m in length, a subsequent paper suggested that the original estimates were reasonable (Mollet et al., 1996). Specifically, the authors used a variety of total length estimation equations based on various other morphometric measurements (e.g., pectoral fin height) to validate the previous estimates. Overall, length estimates for KANGA ranged from 5.3 to 8.2 m and for MALTA between 4.6 and 7.0 m (Mollet et al., 1996). We also note that an individual of reported 8 m total length was caught off Mallorca in 1969 but other estimates from L–W relationships and photographs indicate the individual was 6.00–6.42 m (Morey et al., 2003).

Lamnid sharks, including *C. carcharias*, are unique among sharks in that they can maintain a body temperature above that of their surrounding environment (Dickson & Graham, 2004). They accomplish this by having a capillary network between their swimming muscles, and by continuously swimming can use muscle energy to heat the body (Goldman, 1997; Bernal et al., 2001). *Carcharodon carcharias* subsists on blubber stores from mammals, and can survive on a single, high-calorie meal, typically a marine mammal, for about a month and use these stores to fuel long-distance migration and to help maintain buoyancy (Carey et al., 1982) . If *C. carcharias* were larger, the metabolic demand would require greater scavenging of marine mammals, rare in many oceans (Carey et al., 1982), or increased predation rates on live marine mammals potentially requiring greater metabolic expenditure for prey capture and manipulation.

Body size estimates for *C. carcharias* in our dataset ranged from 0.35 m to 7.13 m, and were normally distributed with a mean of 3.81 m (Fig. 15). Suprisingly, popular media estimates of *C. carcharias* total length were comparable: min = 1.22 m, max = 7.62 m, mean = 4.36 m, and were normally distributed (Fig. 15), although the two distributions were significantly different from one another (Kolmogorov–Smirnov Test: *p* = 0.002). Interestingly, we found that differences in total length estimates depended on the type of “encounter” that *C. carcharias* had with a human (*p* = 8.7 × 10^−5^, Fig. 16). For example, fatal attacks on average were purportedly by larger individuals (mean = 5.03 m), while non-fatal attacks were by smaller individuals (mean = 3.90 m). This could possibly be because learning occurs in the predatory behavior of great white sharks, as great white sharks are known to change their prey throughout ontogeny (Estrada et al., 2006). Alternatively, and perhaps more realistically, there may be a direct relationship between observer perception of shark body size and attack severity. After all, a story about a puny shark inflicting damage is not that impressive.

**Figure 18:**
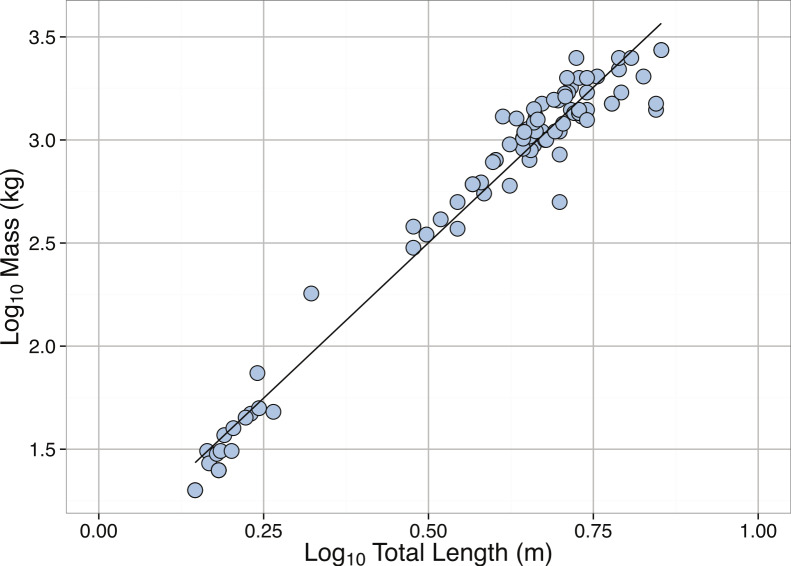
Linear regression between **Log_10_** Total Length (m) and **Log_10_** Mass (kg) for *Carcharadon carcharias*. See Table 2 for regression equations.

**Figure 19:**
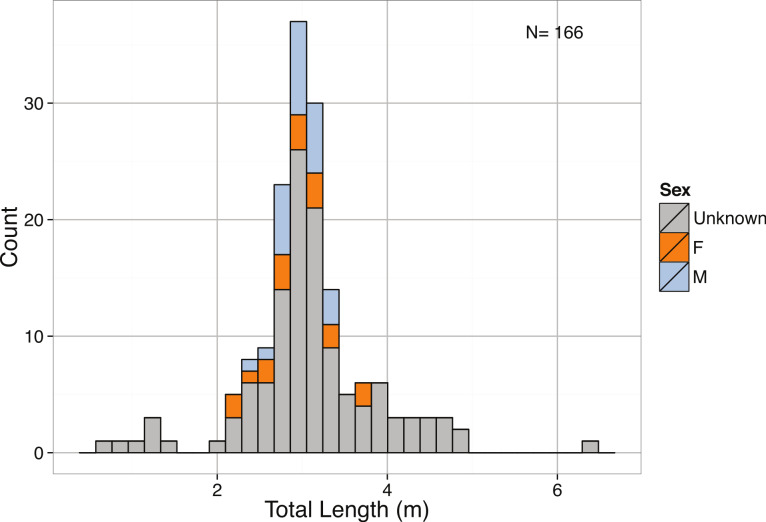
Distribution of Total Length (m) of mature *Somniosus microcephalus* by sex.

We found a significant difference in body length between sexes, with the males being shorter than the females (Female: mean = 4.03 m, *N* = 252, Male: mean = 3.60 m, *N* = 194, *t*-Test: *p* < 0.001), although this finding has not been consistently supported by previous studies (Jorgensen et al., 2010). We also found that shark lengths differed between hemispheres, with individuals in the Southern Hemisphere being smaller on average than individuals in the Northern Hemisphere (Southern: mean = 3.38 m, Northern: mean = 4.18 m, *t*-Test: *p* < 0.001; Fig. 17). Mean shark lengths also differed between ocean basins (Indian: 4.69 m, Mediterranean: 4.51 m, Atlantic: 4.23 m, Pacific: 3.77 m, Southern: 3.33 m, *p* < 0.001; Fig. 17). However, this variation could be due to differences in collection method.

**Figure 20:**
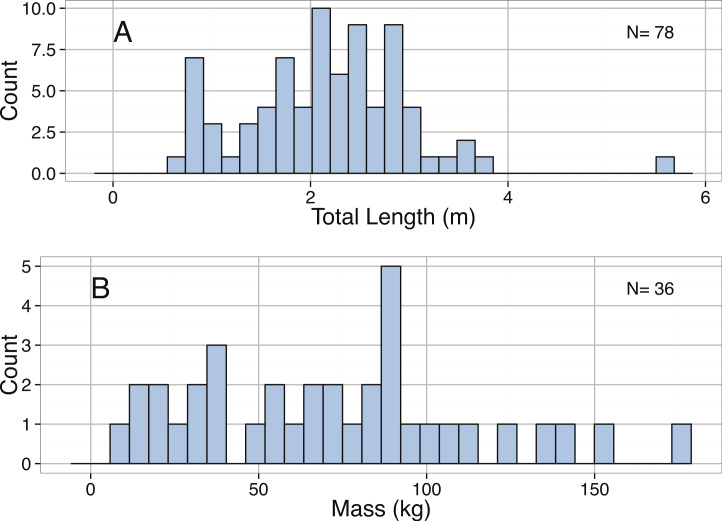
Distribution of (A) Total Length (m) and (B) Mass (kg) of mature *Hexanchus griseus*.

Consistent with previous findings (Klimley & Ainley, 1998), we found a significant relationship between total length (m) and mass (kg) in adult *C. carcharias* (Table 2, Fig. 18). However, other work has found larger slopes and intercepts (Godfried, Compagno & Bowman, 1996).

**Figure 21:**
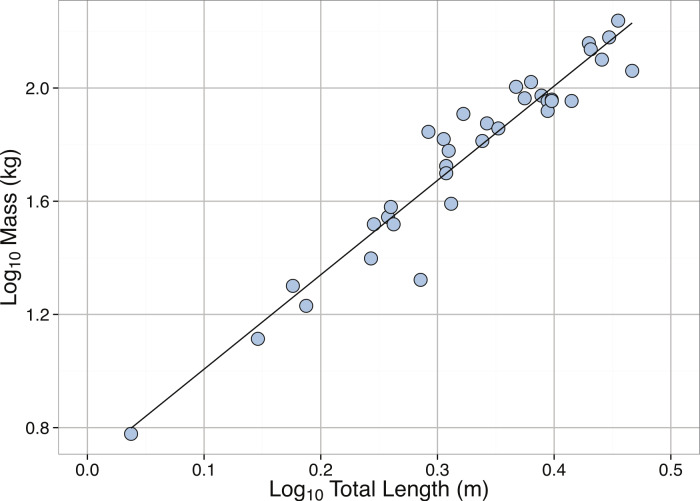
Linear regression between **Log_10_** Total Length (m) and **Log_10_** Mass (kg) for *Hexanchus griseus*. See Table 2 for regression equations.

### Largest Arctic Selachimorphan: Greenland Shark, *Somniosus microcephalus* (Bloch & Schneider, 1801)

The Greenland shark, *Somniosus microcephalus*, is in the family Somniosidae, commonly known as the sleeper sharks, and is one of only two Arctic-dwelling sharks, living in water temperatures around 0.6–12 °C (Ebert, Fowler & Compagno, 2013). Little is known about the basic ecology of the Greenland Shark, as prey consumption rates, metabolic rate, and other life-history parameters are still poorly known (MacNeil et al., 2012). *Somniosus microcephalus* is an opportunistic feeder (Frisk et al., 2002) that scavenges and preys primarily on benthic species, including various macro-invertebrates, fish, and marine mammals (MacNeil et al., 2012; Davis et al., 2013), and are thus typically found at depths down to 1,200 m, although there has been a report of a large male at 2,200 m. Greenland Sharks are found in deeper water at lower latitudes, and have been documented in the Gulf of Mexico where a 3.65 m long specimen was collected from a depth of approximately 1,800 m in 2014. *Somniosus microcephalus* is a slow-living species and is ranked as the slowest swimming fish (Watababe et al., 2012), grows 0.5–1.1 cm per year (Hansen, 1963), and can possibly live to 100–150 years (Ebert, Fowler & Compagno, 2013). The maximum size reported for the Greenland shark is 6.4 m in length, making *S. microcephalus* one of the largest extant shark species (Davis et al., 2013).

The minimum, maximum, and mean reported lengths for *S. microcephalus* that we found were 0.68, 6.4, and 3.07 m, respectively (Fig. 19). The majority of length measurements were much smaller than the maximum reported length (Quantiles: 75% 3.31 m, 90% 3.96 m, and 95% 4.45 m); however, the distribution of sizes was not significantly different from normal (Table 3). Although it has been reported in the literature that females tend to be larger than males, we found no significant difference in mean body length between sexes (Male: 2.96 m, Female: 2.93 m, *p* = 0.77; Fig. 19).

**Figure 22:**
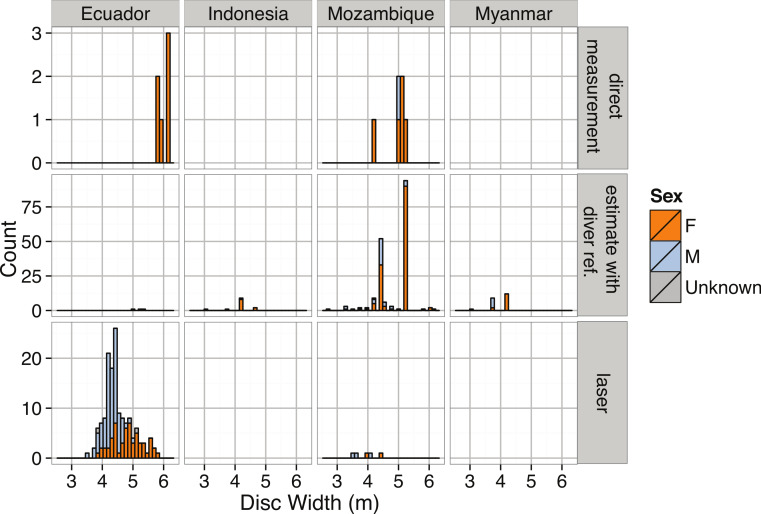
Distribution of Disc Width (m) for *Manta birostris* by measurement method, sex, and region.

**Figure 23:**
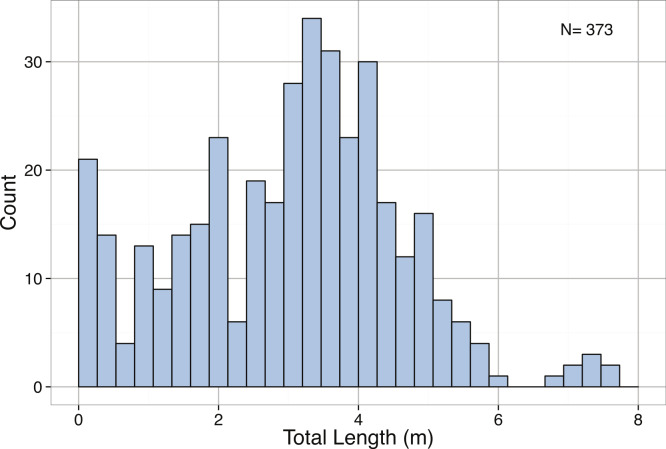
Distribution of Total Length (m) for *Regalecus glesne*.

### Largest Hexanchoid Selachimorphan: Bluntnose Sixgill Shark, *Hexanchus griseus* (Bonnaterre, 1788)

The hexanchoid sharks, or cow sharks, are similar to Greenland Sharks in being deep-water sharks. While *H. griseus* has a global distribution, we were only able to obtain data from a small portion of their full range: from the Pacific and Mediterranean. Several scientific and public websites give maximum length of *H. griseus* as 4.82 m (Compagno, 1984; Ebert, Fowler & Compagno, 2013), which we were unable to confirm. Regardless, the largest recorded length in our dataset is 5.5 m (Celona, De Maddalena & Romeo, 2005).

Deep-water sharks have special adaptations to maintain neutral buoyancy while under pressure (Whetherbee & Nichols, 2000). An adaptation to deep waters is of low-density oil in large livers; the density of oil changes throughout ontogeny and scales with body size (Whetherbee & Nichols, 2000). Increases in body size may thus require changes in the proportions of low-density liver oils, which may be potentially expensive to maintain, particularly in a more nutrient poor environment. *Hexanchus griseus* dives to at least 2,500 m (Ebert, Fowler & Compagno, 2013) and is occasionally seen in the pelagic zone (Carey & Clarke, 1995), but is usually found between 600 and 1,100 m depths (Carey & Clarke, 1995).

Both the total length (TL) and body mass estimates for *H. griseus* are normally distributed (Table 3; Fig. 20). We also found a significant relationship between *H. griseus* total length (m) and body mass (kg) (Table 2; Fig. 21).

**Figure 24:**
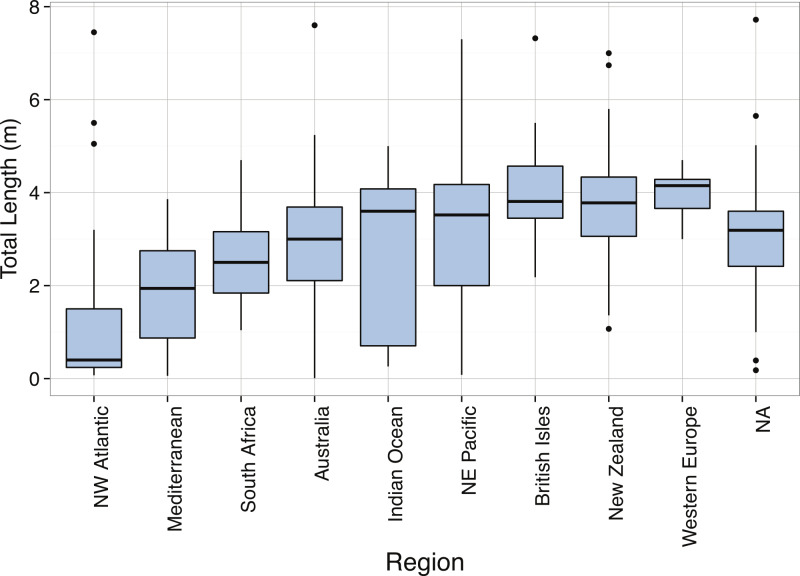
Boxplots of Disc Width (m) for *Regalecus glesne* by region.

**Figure 25:**
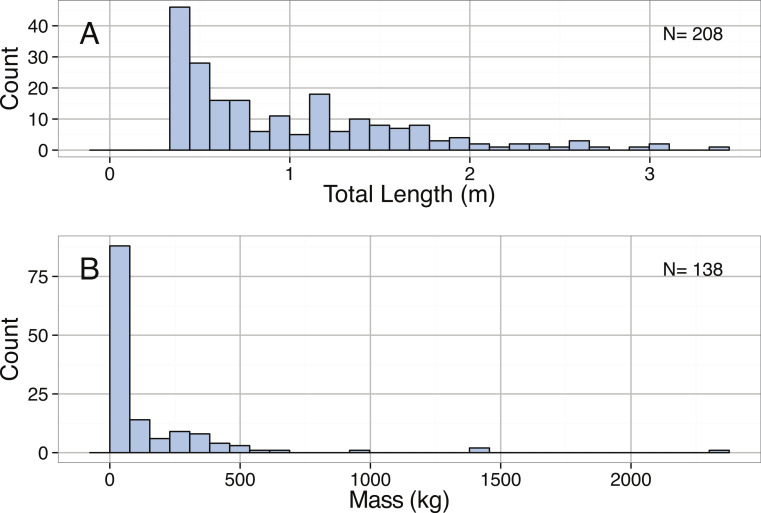
Distribution of (A) Total Length (m) and (B) Mass (kg) for *Mola mola*.

### Largest Batoidean: Giant Ocean Manta Ray, *Manta birostris* (Walbaum, 1792)

Manta rays are the largest batoids in the world, with two recognized species: *Manta birostris* and *Manta alfredi*. *Manta birostris* exhibits a broad distribution, occurring in tropical, sub-tropical and temperate waters around the globe. These planktivorous fish are commonly sighted along productive coastlines with regular upwelling or oceanic island groups, offshore pinnacles and seamounts (Compagno, 1999; Marshall, Compagno & Bennett, 2009; Couturier et al., 2012). *Manta birostris* has been documented to occur as far north as southern California and Rhode Island on the United States west and east coasts; Mutsu Bay, Aomori, Japan, the Sinai Peninsula, Egypt and the Azores Islands in the Northern Hemisphere; and as far south as Peru, Uruguay, South Africa and Tasmania in the Southern Hemisphere (Marshall, Compagno & Bennett, 2009). Dozens of major aggregation sites for *M. birostris* have been identified worldwide, although the frequency and the abundance with which they are observed can vary dramatically (Kashiwagi et al., 2011; Marshall et al., 2011).

The giant manta ray, *M. birostris,* reaches disc widths (DW) of at least 7 m (Newman, 1849; Bigelow & Schroeder, 1953) with anecdotal reports up to 9.1 m DW (Alava et al., 1997; Compagno, 1999), and can weigh up to 2,721 kg (Coles, 1916). The largest reported specimen in our dataset has a disc width of 6.2 m. The distribution of disc widths is not significantly different from normal for the combined regional datasets (Table 3, Fig. 22). However, when considered separately, the size distributions for Ecuador and Mozambique are significantly right- and left-skewed, respectively (Table 3). *Manta birostris* is sexually dimorphic, with female rays reaching maturity at larger overall disc widths than males and ultimately achieving larger maximum sizes as well (Marshall & Bennett, 2010) (Fig. 23).

**Figure 26:**
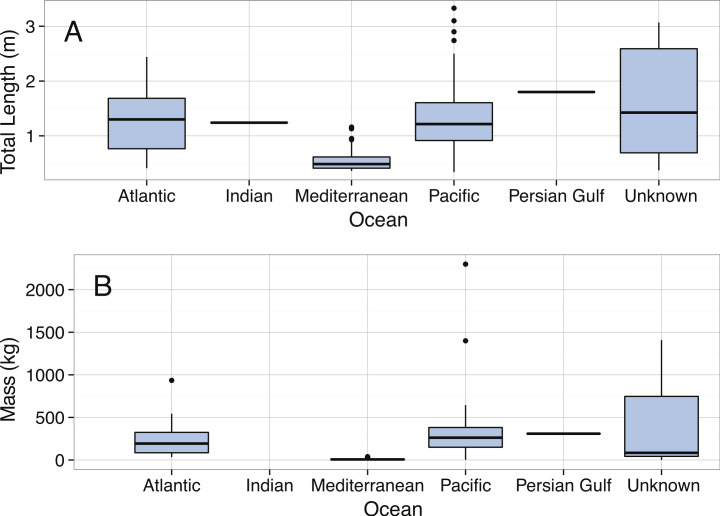
Boxplots of (A) Total Length (m) and (B) Mass (kg) for *Mola mola* by Ocean.

**Figure 27:**
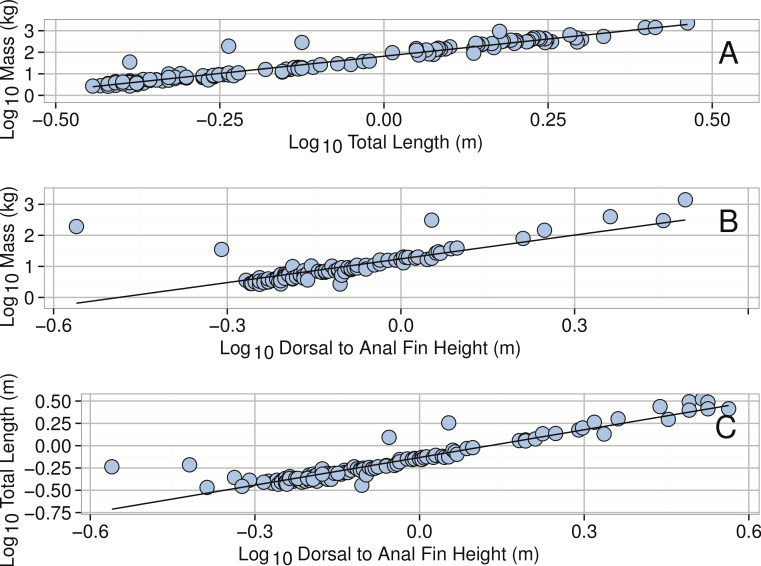
Linear regressions for *Mola mola*. (A) Log_10_ Total Length (m) and Log_10_ Mass (kg). (B) Log_10_ Doral to Anal Fin Height (m) and Log_10_ Mass (kg). (C) Log_10_ Doral to Anal Fin Height (m) and Log_10_ Total Length (m). See Table 2 for regression equations.

Size estimates are often achieved by comparing these large rays to subjects of known size, e.g., reference to a diver’s length. Alternatively, laser photogrammetry, where measurements are extrapolated from a distortion-corrected photograph of a subject onto which twin parallel laser dots of a known spacing have been projected, is currently one of the most reliable methods of size estimation (Deakos, 2010). Recent attempts to study the size range of manta rays has revealed that different populations of *M. birostris* also show geographic variability in average and maximum observed disc width sizes (Fig. 23). These differences may be directly related to differences in food availability between regions or could be a function of human-induced pressure on populations. However, we caution that differences may reflect uneven sampling across different regions or differences in methodologies. For example, laser estimates largely dominate the size data from Mozambique. However, a model accounting for measurement method stills yields significant differences in size among regions (*p* = < 2 × 10^−16^–7.72 × 10^−06^).

**Figure 28:**
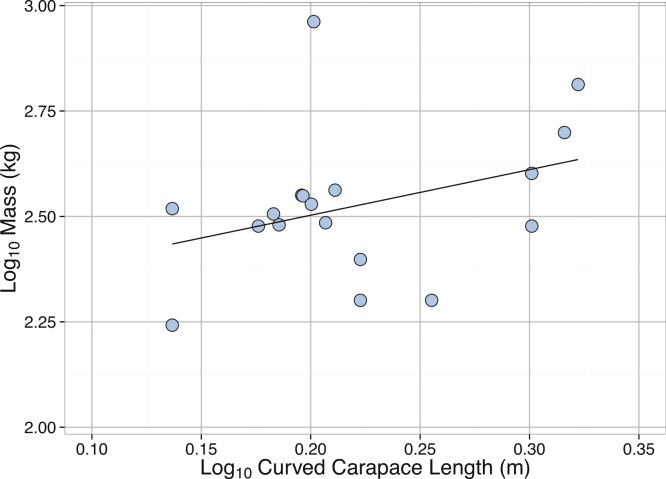
Relationship between **Log_10_** Total Length (m) and **Log_10_** Mass (kg) for *Dermochelys coriacea*. See Table 2 for regression equations.

Manta rays have very conservative life histories and are considered to be some of the least fecund of all elasmobranch species, with extremely low reproductive outputs, making them vulnerable to overfishing (Ward-Paige, Davis & Worm, 2013; Dulvy et al., 2014). Unfortunately, both species have a high value in international trade, and direct fisheries exist that target these species in unsustainable numbers (Couturier et al., 2012; Ward-Paige, Davis & Worm, 2013). The escalating fishing pressure on manta rays globally resulted in the elevation of the status of *M. birostris* to Vulnerable on the IUCN Red List of Threatened Species in 2011 (Marshall et al., 2011). Shortly after, the giant manta was listed on Appendix I and II of CMS (the Convention for Migratory Species Act) in 2011 followed by an Appendix II listing on CITES in 2013. Due to the highly migratory habits of manta rays, targeted fisheries have had broader repercussions throughout the regional distributions of both *Manta* species. In the face of fishing pressure and other anthropogenic threats, it is likely that individuals in many populations may not be near their maximum possible ages or sizes.

### Longest Osteichthyan: Oarfish, *Regalecus glesne* (Ascanius, 1772)

Although several species have been erected in the genus *Regalecus*, morphometrics suggests that two valid species occur, *R. glesne* and *R. russellii*, both with cosmopolitan distributions, and attaining similar maximum sizes of approximately 8 m in length (Roberts, 2012). Although reports of *R. glesne* reaching lengths greater than 8 m in total length exist, these are very likely inaccurate estimates and measurements. From Roberts (2012) recent thorough description of *Regalecus,*

*“The largest specimens preserved in museum collections, of both R. russellii and R. glesne, are just under 8 m total length. I have not been able to find any evidence that Regalecus ever attain lengths greater than this. The report of a 15–16 m (50–60 foot) long Regalecus stranded on Stronsay Island in the Orkneys in 1808 is based upon the rotting carcass of a large basking shark. Records of oarfish 10, 10.7, or 11 m total length are based on addition of extrapolated lengths of the posterior part of the body lost at much smaller sizes.”*

Roberts (2012) provided comprehensive and verified length measurements for *R. glesne*, which we analyzed here. The size distribution was bimodal, with a distinctive peak at juvenile lengths and at 4 m adult lengths (Fig. 23). Most specimens were well below 5 m long. The distribution of adult lengths was normally distributed (Table 3).

**Figure 29:**
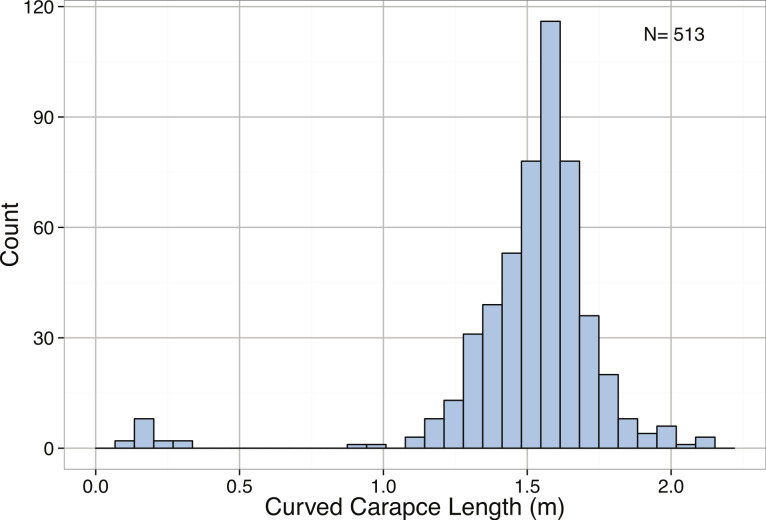
Distribution of Curved Carapace Length (m) for *Dermochelys coriacea*.

Some geographic variation in size does appear in *R. glesne* (Fig. 24). Mean lengths in the Northwest Atlantic, Mediterranean, and South Africa were much shorter relative to those reported from Western Europe, British Isles, Scandinavia, and New Zealand, and may be consistent with Bergmann’s Rule (Bergmann, 1847).

**Figure 30:**
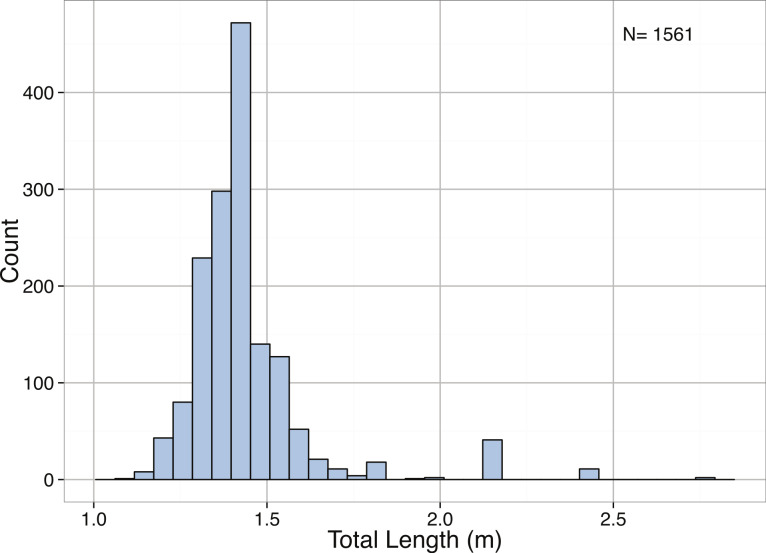
Distribution of Total Length (m) for *Mirounga leonine*.

Oarfish are oceanic fishes that normally inhabit the epipelagic and upper mesopelagic zones (Roberts, 2012). Encounters with healthy animals in the open ocean are rare (Benfield et al., 2013). It is also important to note that almost all size measurements of *R. glesne* and *R. russellii* are derived from dead or dying animals that have washed up on shorelines, or been stranded in shallow, coastal waters. Consequently, our estimates of the maximum length attainable by either species are based on measurements of individuals that were not randomly collected from a healthy population. Given how little we know about oarfish, one can only speculate about what constraints there may be to attaining their maximum size.

### Heaviest Osteichthyan: Ocean Sunfish, *Mola mola* (Linnaeus, 1758)

*Mola mola* is a globally distributed species. The largest recorded size for *M. mola* is 3.3 m in length, 3.2 m in height, and 2,300 kg from an individual that washed ashore at Whangarei Heads in New Zealand (Rowan, 2006). Another potential candidate for the largest was a specimen caught off the coast of Kamaogawa, Japan in 1996 in set nets owned and operated by the Kamogawa Fisheries Cooperative Association. This specimen measured 2.7 m in length and weighed 2,300 kg (Roach, 2003). The difficulty of obtaining lengths and masses of large live individuals while at sea has likely resulted in the omission of large specimens from other oceans.

Freedman & Noakes’ (2002) survey on the limits of size in teleosts and elasmobranchs discussed several factors that could limit teleost size. Specifically, they focused on four major areas: anatomical, physiological, ecological, and life-history constraints. They argued that the only viable candidate limiting the size of bony fish is the size of gills and the requirement to pump water over them for ventilation. This factor, combined with the higher metabolic demand of teleosts verses elasmobranchs, provides some indication why *M. mola* does not reach the size of the largest elasmobranchs. Indeed, *M. mola* is relatively sluggish compared to other fish, giving further credence to this hypothesis (Freedman & Noakes, 2002). Freedman & Noakes (2002) also noted that the next largest fishes, i.e., tunas and marlins, augment respiration with ram-jet ventilation.

The distributions of lengths and masses for *M. mola* were considerably right-skewed (Fig. 25) and both distributions were significantly different from normal (Table 3). A majority of the lengths and masses were far less than that of record holders. The similar sizes of *M. mola* found in the Atlantic and Pacific Oceans suggest that, as a globally distributed species, *M. mola* does not demonstrate large geographic variations (Fig. 26).

**Figure 31:**
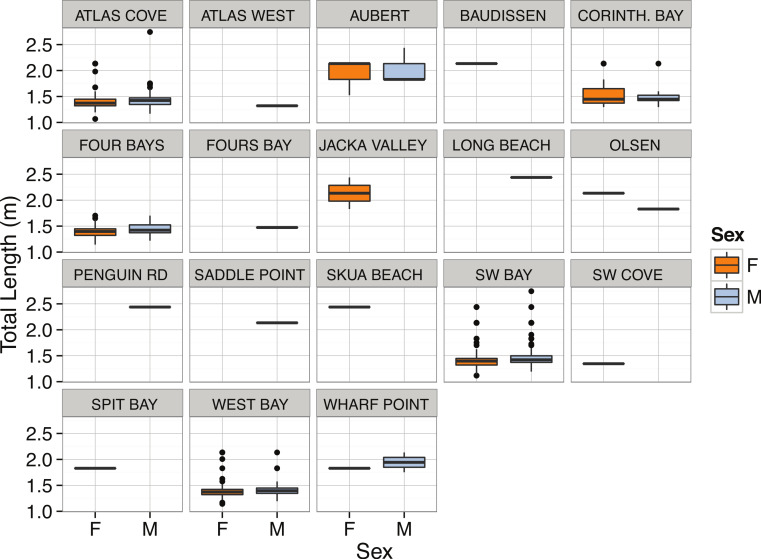
Boxplots of Total Length (m) and for *Mirounga leonine* divided by sex and location.

**Figure 32:**
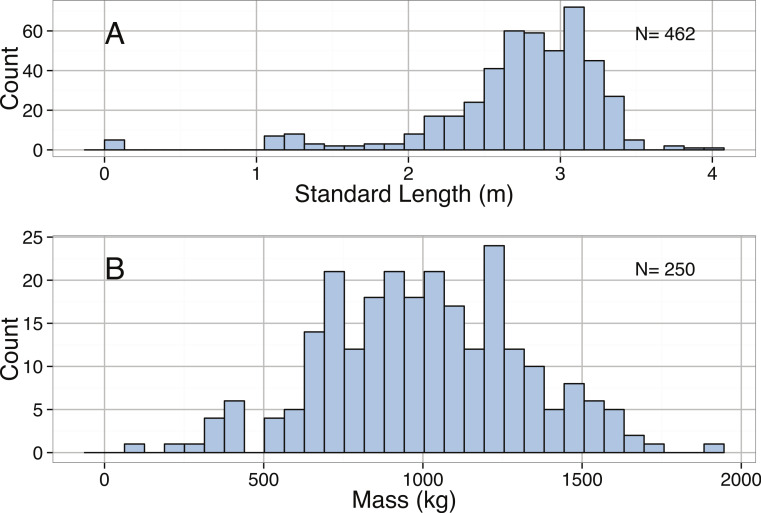
Distribution of (A) Total Length (m) and (B) Mass (kg) for *Odobenus rosmarus*.

A significant allometric relationship (Fig. 27) exists between total length and body mass and between dorsal to anal fin (DAF) length and body mass (Table 2). Dorsal to anal fin length is also a significant predictor of total length (Table 2).

**Figure 33:**
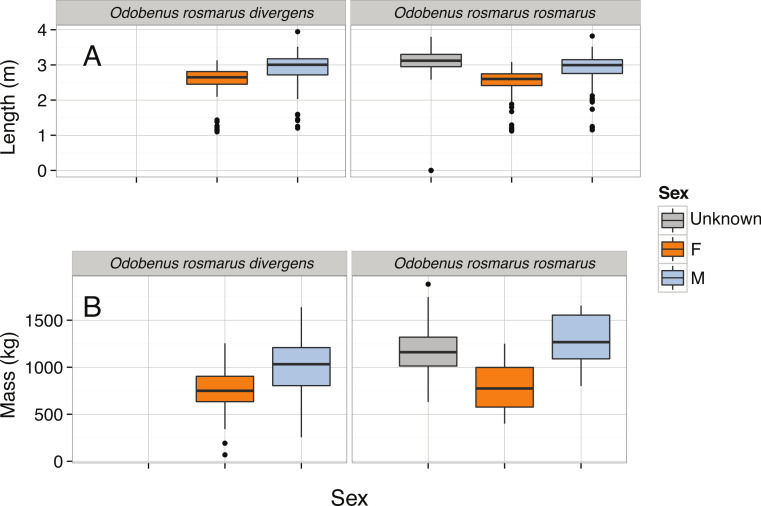
Boxplots of (A) Total Length (m) and (B) Mass (kg) for *Odobenus rosmarus* divided by subspecies and sex.

### Largest Testudines: Leatherback Turtle, *Dermochelys coriacea* (Vandelli, 1761)

*Dermochelys coriacea* is the largest of all sea turtles and the fourth largest living reptile. *Dermochelys coriacea* is well known for long migrations and deep dives, and is the only sea turtle commonly found in cold waters. However, only large adult leatherbacks are observed in cold temperate waters and little is known about distribution and migration patterns of juveniles, as they have rarely been observed between hatchling and adult sizes (Shillinger et al., 2012). The little data available suggests that juveniles are more commonly observed in warmer waters (Eckert, 2002), possibly due to a lack of ability to maintain sufficiently elevated temperatures due to thier small size.

The largest leatherback turtle in terms of curved carapace length (CCL), a standard metric for *D. coriacea* size, is 2.13 m. This Arabian Sea male was stranded on the Pakistani beach of Sanspit (Firdous, 1989). The largest known leatherback by mass is 916 kg. This particular turtle is referred to as the Harlech turtle (after the beach in Wales where it was discovered) and is purported to be the largest leatherback ever found (Davenport, Holland & East, 1990). However, our inspection of the relationship between CCL and mass suggests the Harlech turtle could be anomalous (Fig. 28). Compared to other measured *D. coriacea* with similar CCLs of ∼1.5 m, masses are just one-third of the Harlech turtle’s mass at ∼300 kg. The largest mass in our dataset after the Harlech turtle is a 650 kg specimen with a CCL of 2.1 m. A number of explanations may account for the Harlech turtle discrepancy. First, our allometric equation (Table 2) does not accurately describe the relationship between turtle CCL and mass due to a low sample size (*N* = 16) and relatively poor fit (*p* = 0.0330, *R*^2^ = 0.22). Second, our simple model did not consider other factors that may explain much of the variation in turtle sizes such as age, sexual dimorphism, and geography. For example, males in our dataset were larger than females with respect to CCL (*p* = 1.63 × 10^−8^, *R*^2^ = 0.64, Female Mean CCL = 1.57 m, Male Mean CCL = 1.62 m). More likely, however, the mass estimate for the Harlech turtle is simply inaccurate. We have confirmed the CCL measurement with the National Museum Cardiff that currently houses the specimen; however, mass was measured at the time the turtle was originally stranded in 1988 and details of the measurements cannot be ascertained.

**Figure 34:**
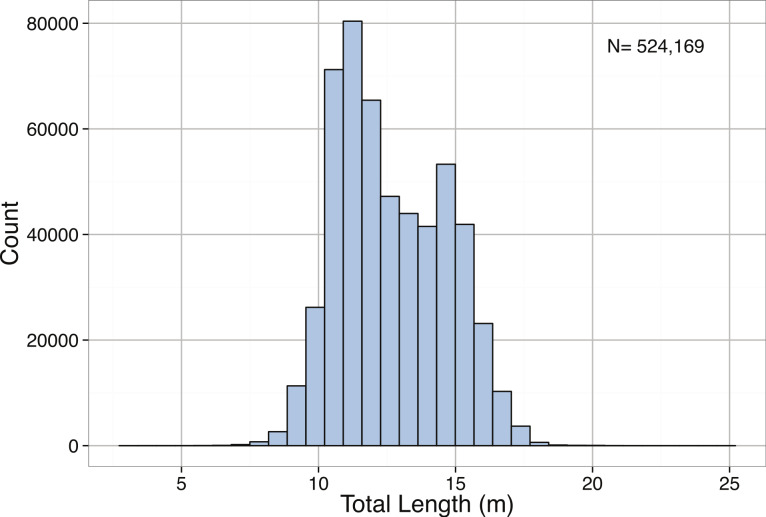
Distribution of Total Length (m) for *Physeter macrocephalus*.

The upper and lower limits to size for leatherbacks likely stem from thermal and nutritional pressures. *Dermochelys coriacea* likely experiences strong selection pressure for efficient thermoregulation, and spends time in waters as cold as 0.4 °C while diving (Davenport, Holland & East, 1990). Their large size allows them to possess some characteristics of endothermy, enabling the maintenance of high core body temperatures in cold waters (Davenport, Holland & East, 1990). A comparative study on the metabolic rates of leatherbacks relative to other reptiles found that *D. coriacea* are able to maintain elevated body temperatures in cold waters despite having a low metabolic rate, for which the term gigantothermy was suggested (Paladino, O’Connor & Spotila, 1990). The large size of this species allows for a low surface area to volume ratio to minimize heat loss, as well as room for extensive blubber under a leathery skin (Davenport, Holland & East, 1990). This is also combined with circulatory counter-current heat exchangers that increase internal temperatures further (Paladino, O’Connor & Spotila, 1990).

The upper size of *D. coriacea* may be limited by caloric restrictions, as their prey consists of gelatinous medusae with low nutritional value. One theoretical model has shown that a 300 kg leatherback would need to feed for 3–4 h a day to meet its minimum energetic requirements, even if their jellyfish prey occur in dense patches (Fossette et al., 2012). Fossette et al. (2012) also suggested that a high encounter rate between predator and prey in this case is crucial for leatherbacks to be able to sustain themselves on this type of food source. Recent observations of leatherback turtles foraging among very high densities of lion’s mane jellyfish (*Cyanea capillata*) and moon jellyfish (*Aurelia aurita*) off California demonstrated that such high encounter rates were possible and turtles consumed an average of 330 kg wet mass d^−1^ (66,018 kJ d^−1^), an amount that was estimated to be 3–7 times their daily metabolic energy requirement (Healslip et al., 2012).

We found that most curved carapace lengths were far below the record holder CCL of 2.13 m. The distribution of CCLs was considerably left-skewed (Table 3, Fig. 29). There was a clear gap in the data between lengths of 0.3 m and 0.9 m, which corresponds to subadults and juveniles. Although it is possible that this gap may be explained by our small sample size, it is fairly interesting that it is consistent with the general lack of knowledge of what happens to these leatherbacks at the ages corresponding to these sizes (Shillinger et al., 2012). A removal of hatchlings and small juveniles from the dataset yielded a normal distribution (Table 3).

**Figure 35:**
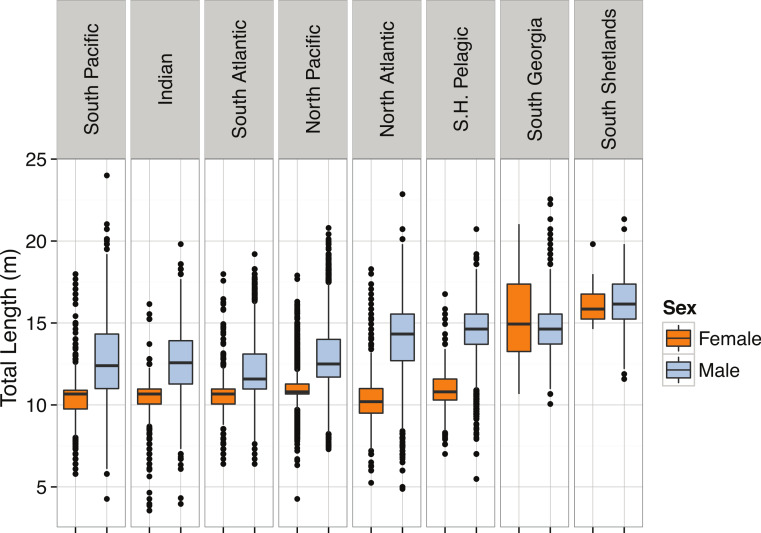
Boxplots of Total Length (m) by region and sex for *Physeter macrocephalus*.

The clearest issue limiting our understanding of geographic variation in size, sexual dimorphism, and potential decreases in size over time is a lack of data. Although there are several stranding networks for *D. coriacea*, there is no single, integrated, open-access database. Some online-access databases only report nesting counts for leatherbacks, and do not make size measurements available. Additionally, individual stranding networks appear to operate autonomously, with little coordination of efforts or collaborative data sharing. There is a clear need for more collaboration on data regarding this endangered organism.

### Largest Pinniped and Carnivoran: Southern Elephant Seal, *Mirounga leonina* (Linnaeus, 1758)

Elephant seals of the genus *Mirounga* are found in the northeastern Pacific Ocean (northern elephant seals, *M. angustirostris*) and in the sub-Antarctic and Antarctic oceans (southern elephant seals, *M. leonina*). The southern elephant seal is the largest carnivoran—even larger than the semi-aquatic polar bear, *Ursus maritimus*. *Mirounga leonina* shifts its distribution and diet within its geographic range depending on the season, feeding on fish around the Antarctic shelf in winter, and on large species of pelagic squid in the summer (Slip, 1995; Bradshaw et al., 2003).

The Guinness Book record holders for maximum size for *M. leonina* are a male that measured 6.85 m long and weighed 5,000 kg, and a 3.7 m, 1,000 kg female (Wood, 1982). For *M. angustirostris*, the maximum reported size for a male is 4 m and 2,300 kg, and for a female 3 m and 640 kg (Wood, 1982). The large size of elephant seals might confer a foraging benefit. The ability of these seals to follow vertically moving prey was positively related to mass, with smaller seals having shorter dive durations and shallower dive depths (Irvine et al., 2000). Large size may also reduce predation risk both in and out of the water for the species.

*Mirounga leonina* exhibits extreme sexual dimorphism, where males are significantly larger than females (Bryden, 1972; Galimberti et al., 2007) Currently, there are two hypotheses for why males are larger: (1) males fast during the reproductive season to secure their harem, and therefore must acquire considerably more blubber than females (Galimberti et al., 2007), and (2) males engage in male–male fighting to gain possession over harems (McCann, 1981; Galimberti et al., 2007). Interestingly, females need to reach a minimum body mass, 300 kg, before they are able to reproduce (Arnbom, Fedak & Rothery, 1994). Further, only above 380 kg will females begin giving birth to males (Arnbom, Fedak & Rothery, 1994); typically male pups are larger than female pups (McCann, Fedak & Harwood, 1989).

The majority of the *M. leonina* lengths reported—for both males and females combined—are less than 1.4 m in length (Fig. 30). Females were slightly smaller than males (Females: median = 1.39 m; Males: 1.42 m; *p*-value = 0.00295, Fig. 31). The small differences in size between sexes may reflect the use of length as opposed to mass. To date, there are not any papers relating total body length to body mass except for Bryden (1972). We also observed considerable variation in body length between sites (*p* < 2.2e^−16^, Fig. 31).

**Figure 36:**
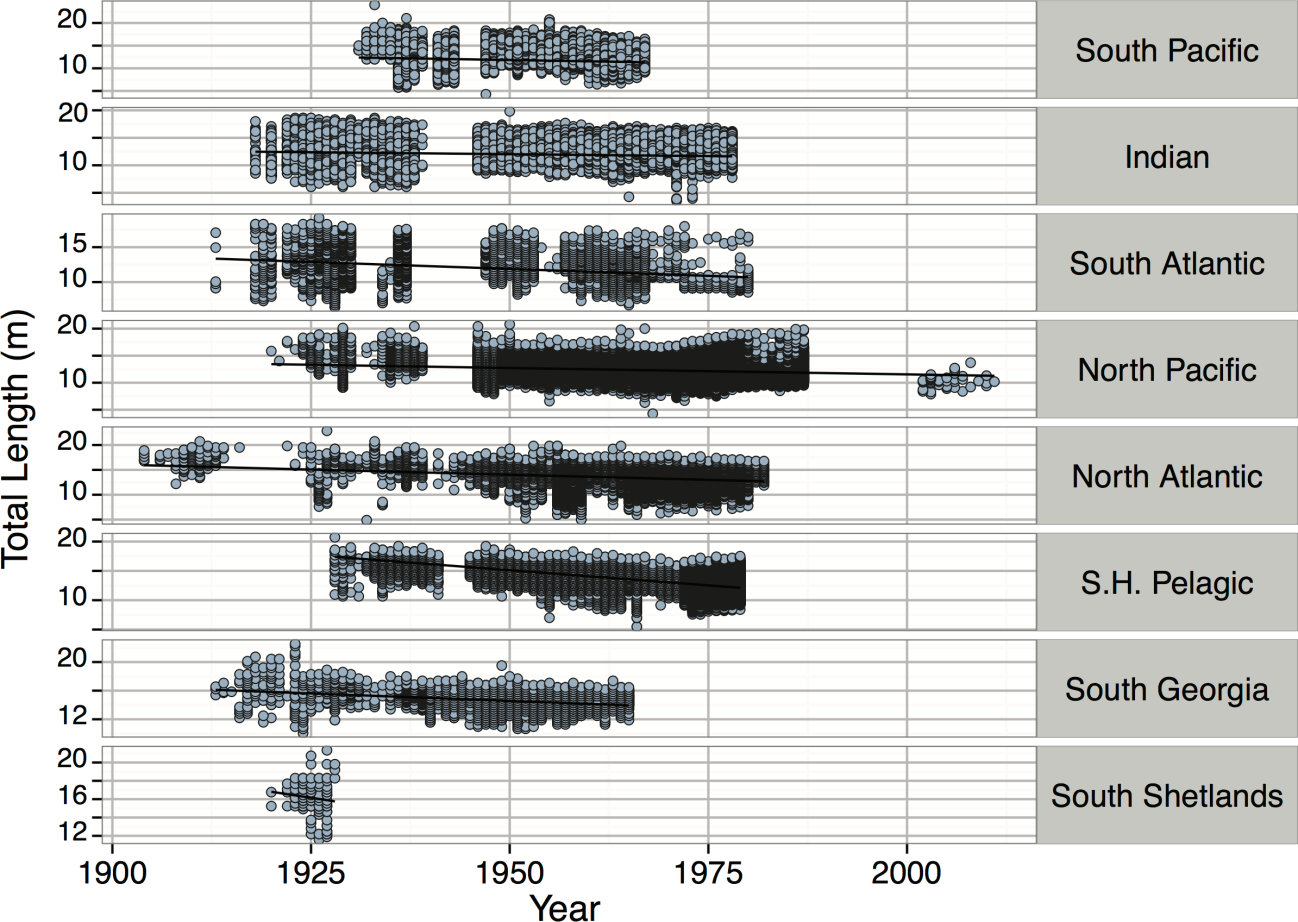
Total Length (m) versus year by region for *Physeter macrocephalus*.

**Figure 37:**
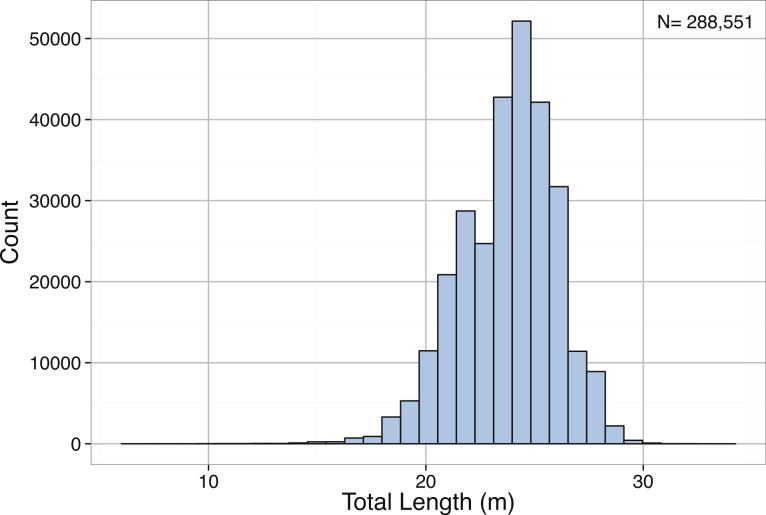
Distribution of Total Length (m) for *Balaenoptera musculus*.

### Third Largest Pinniped: Walrus, *Odobenus rosmarus* (Linnaeus, 1758)

*Odobenus rosmarus* has a disjunct distribution occurring across the Arctic Ocean and sub-Arctic portions of the North Pacific and North Atlantic. There are three recognized subspecies: the Atlantic Walrus *O. r. rosmarus,* the Pacific Walrus *O. r. divergens* (Illiger, 1811), and the Laptev Walrus *O. r. laptevi* (Chapskii, 1940). The Laptev Walrus taxonomic status is questionable as prior morphological work (Fay, 1981), and more recent DNA work, suggests that this subspecies is simply a western continuation of the Pacific Walrus and the name should be discontinued (Lindqvist et al., 2009). The IUCN classification for *O. r. divergens* is currently listed as data deficient.

For *O. rosmarus*, the largest recorded specimen is predicted to be 2,500 kg based on a hide weight 500 kg and the assumption that the hide compromises roughly 20% of total weight (Wood, 1982). Captain Ole Hansen killed this individual in 1909 near Franz-Josef Land. In 1911, the American Jack Woodson shot a bull with a purported weight of 2,268 kg (Wood, 1982). Both of these records are difficult to verify. The largest walrus we can confirm is an *O. r. rosumarus* male from southeastern Svalbard, a Norwegian archipelago in the Arctic Ocean, that measured 1,883 kg and was 3.8 m long (Wiig & Gjertz, 1996). Wiig & Gjertz (1996) did note, however, that this size estimate may be conservative: “If we take into account that the standard length might be too short, and the possible underestimation of weight based on Eq. (1) of Knutsen & Born (1993); it is believed that the largest male walruses at Svalbard might weigh about 2,000 kg.”

Reproductive rates of *O. rosmarus* are the lowest of any pinniped, with gestation occurring for 15 months and nursing of calves for more than a year thereafter (Garlich-Miller & Stewart, 1999). Females may spend 2–3 years before resuming calving (Garlich-Miller & Stewart, 1999), likely needed to rebuild energy reserves. Increases in maximum size would further increase this reproductive period, potentially lowering reproductive rates below those needed to offset mortality rates. Likewise, increases in maximum size may also increase fetal and calf growth rates, further increasing metabolic demand on females. The selection for larger sizes, among many pinnipeds, may reflect an adaptation to predation pressure when on land. The strong sexual dimorphism, with males being considerable larger, likely reflects selection for dominance in social interactions. Larger male body sizes are equated with increased social dominance and decreases in receiving of tusk strikes and visual threats (Miller, 1975). Agonistic interactions are often directed toward smaller males (Miller, 1975). This social dominance is important, as it leads to greater harem sizes and presumably greater fitness for larger males (Linderfors, Tullberg & Biuw, 2002).

Maximum sizes were clearly skewed toward larger lengths (Fig. 32). The distribution of standard lengths is left-skewed (Table 3). However, the distribution of masses was normal (Table 3) with quantiles markedly lower than the maximum reported size. Although some prior work (Fay, 1981) has concluded that the standard lengths of Pacific walruses are larger than Atlantic walruses, our data do not support this pattern (Fig. 33). We found no significant difference between the two subspecies in term of standard lengths (*p* = 0.86) after accounting for the significant length differences between males and females (*p* = 0.003). This is similar to patterns previously reported (Knutsen & Born, 1993; McClaren, 1993; Wiig & Gjertz, 1996). We did, however, detect significant differences in the mass between the two subspecies (Fig. 33), with males from the Atlantic being heavier than those in Pacific (*p* = 3.19 × 10^−5^). To a lesser extent, the same pattern also seems to apply to females. Prior work (Knutsen & Born, 1993) found the opposite pattern, with the Pacific subspecies being heavier than walruses from Greenland. Our dataset includes specimens from Svalbard, Norway (Wiig & Gjertz, 1996) which includes several large males. This suggests that the allometric scaling equations between standard length and mass will be different for the two subspecies. Unfortunately, the available data are insufficient to allow a test of this hypothesis because many of the mass measurements were estimated from length measurements.

**Figure 38:**
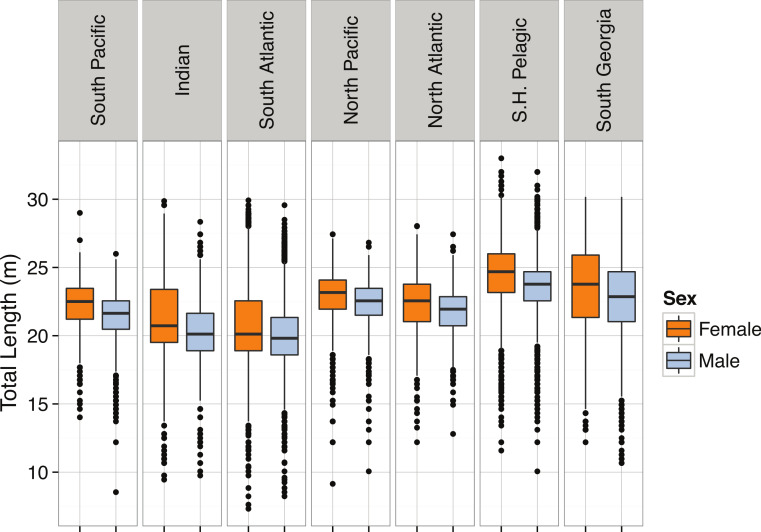
Boxplots of Total Length (m) by region and sex for *Balaenoptera musculus*.

**Figure 39:**
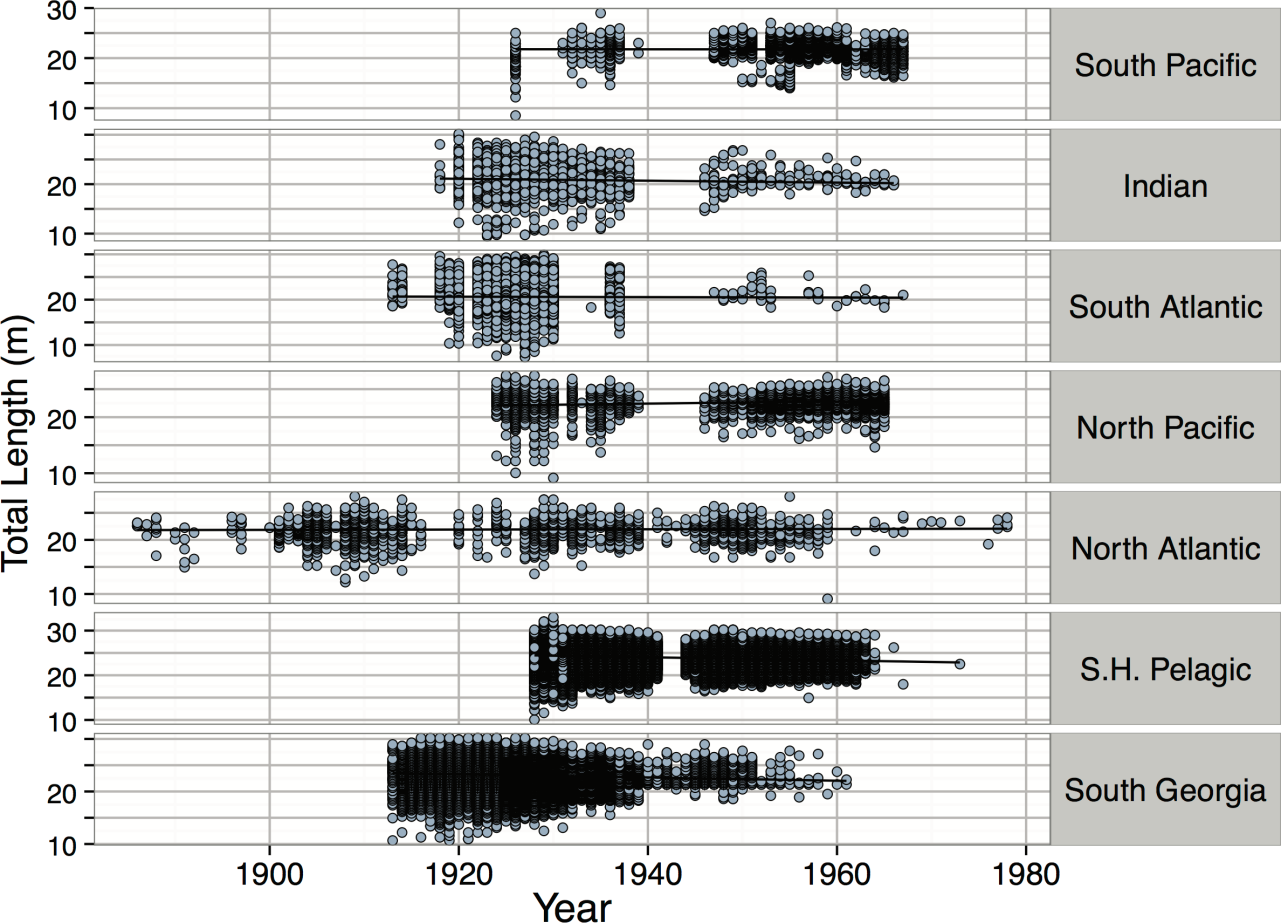
Total Length (m) verses year by region for *Balaenoptera musculus*.

### Largest Odontocete: Sperm Whale, *Physeter macrocephalus* (Linnaeus, 1758)

*Physeter macrocephalus* is the largest of the toothed whales. Sperm whales possess a cosmopolitan geographic distribution but, given their deep diving foraging behavior, tend to occur at depths greater than 1,000 m. The largest known individual, as reported by Guinness Records, was captured off the Kuril Islands in 1950 and measured 20.7 m in length (Carwardine, 1995). However, the jaw of the specimen in the Natural History Museum in London is purported to belong to a 25.6 m long individual (Carwardine, 1995). In our data set the longest measured length is 24 m, given for a male caught in the South Pacific in 1933. Even the next eight largest individuals (22.9–20.8 m) in our dataset are greater than the maximum length reported by Carwardine (1995) and recognized by Guinness Records.

The large size of *P. macrocephalus* may be attributed to their foraging behavior (Evans & Hindell, 2004; Rice, 1989; Watwood et al., 2006; Whitehead, MacLeod & Rodhouse, 2003). As a known deep-sea diver, *P. macrocephalus* regularly dives to depths that few other pelagic animals reach (Watwood et al., 2006). Large size confers a benefit in these deep-sea excursions, not only by making the trip less metabolically costly, but also by increasing aerobic capacity and enabling *P. macrocephalus* to stay submerged for longer periods of time (Watwood et al., 2006; Whitehead, MacLeod & Rodhouse, 2003). Large size also allows *P. macrocephalus* to feed on many cephalopods during a single dive (Rice, 1989). Lindberg & Pyenson (2007) hypothesized that the wide diversity of cephalopods during the Eocene allowed archaeocetes, primitive cetaceans, to exploit mid- and deep-water cephalopods and subsequently evolve into large odontocetes capable of diving to great depths. However, cephalopods may provide a lower quality food source than fish and crustaceans (Evans & Hindell, 2004) and ultimately may energetically limit the maximum size obtainable by odontocetes.

Size data obtained for *P. macrocephalus* came from the International Whaling Commission’s (IWC) whaling records (data held by IWC). Extending as far back as the 1880s, the records detail the ocean basin where individuals were caught, the specific geographical coordinates of capture, the date of capture, and the sex of each individual. The total lengths of individuals were measured from whales at rest on a flat surface, from the apex of the notch between tail flukes to the most forward part of the head. We found that the overall distribution of total lengths for *P. macrocephalus* was right-skewed (Table 3; Fig. 34). Geographic differences in the length of *P. macrocephalus* were found, with individuals in the seas of the Southern Ocean and North Atlantic obtaining larger mean lengths relative to individuals in other regions (Fig. 35). In the North Atlantic and Southern Hemisphere pelagic (open-ocean) whaling, the differences in sizes only occur in males, while at South Georgia and South Shetlands both males and females are larger. The wide variation in the geographic size distribution seen in *P. macrocephalus* is consistent with observations of the whale’s mating patterns and distribution. Individuals found closest to the poles are predominantly solitary large, mature males, while larger groups of females and immature males congregate closer to the equator (Best, 1979).

We also found total length to differ between sexes, with males being larger than females (*p* < 2 × 10^−16^, Fig. 35). *Physeter macrocephalus* are the most sexually dimorphic of the cetaceans, which has been attributed to sexual competition between males for mating opportunities (Rice, 1989; Whitehead, MacLeod & Rodhouse, 2003). Females stop growing at approximately 30 years of age and 10.6 m in length, while males continue growing until they are 50 years old and 16 m long. The sexes also demonstrate differences in geographic distributions, as mature males can be found in waters cooler than 15 °C at the surface, while females and immature males remain in tropical and sub-tropical regions (Best, 1979). Mature males only return to the warmer waters in order to breed (Best, 1979). The mean lengths of males and females were significantly different within all the geographic regions (from *p* = 0.0018 to *p* < 2 × 10^−16^), but relative differences varied between regions. Sexual dimorphism was the weakest among males and females of *P. macrocephalus* caught off South Georgia and South Shetlands, where many mature males and a few large females occur.

In 1999, it was estimated that the population of *P. macrocephalus* was only 32% of the pre-whaling population of 1,100,000 whales (Whitehead, 2002). However, Whitehead’s (2002) estimate of current sperm whale abundance may be too low, since it was assumed that 87% of sperm whales on the survey tracklines were observed (much higher than is realistic, T Branch, pers. comm., 2014). On the other hand, the current population size may be more depleted due to the revelation that the Soviet Union caught many more whales in the 1950s–1970s than they had previously reported (Baker & Clapham, 2004). The disproportionate number of male individuals harvested by whalers (except the later Soviet catches) suggests that they selectively took larger-bodied specimens from which more oil could be produced, a process known as “gunner selection”(Ellis, 2011).

We found a significant temporal decline in the sizes of both males and females (*p* < 2 × 10^−16^, Fig. 36). This trend of decreasing body size over time was consistent for all regions (from *p* = 4.05 × 10^−06^ to *p* < 2 × 10^−16^), with the exception of the South Shetlands (*p* = 0.1932). Declines may be due to heavy whaling on larger individuals, but may also be due to a shift from targeting only large lone males to more indiscriminate targeting of immature males and females, especially during the later period of Soviet whaling (about 1958–1973) when misreporting was rife (Ivashchenko & Clapham, 2014). The lack of pattern in the South Shetlands may be due to relatively lower sample sizes. The only region with an increase in body size was the North Pacific, where the sizes of males caught before 1975 decreased as seen in the other regions, but actually increased from 1975 on. This pattern was probably driven by Soviet whaling in the North Pacific as described above. It has been estimated that the Soviet Union reported only slightly more than half the numbers of their actual catches to the IWC (Ivashchenko, Clapham & Brownell, 2011). Some of the larger whales that were caught were reported to be shorter in order to create a more convincing distribution between the largest whales and the smallest whales, which were reported as longer than they were in order to meet length minimums (Ivashchenko, Clapham & Brownell, 2011). Consequently, once observers were allowed onto whaling ships in 1972 (Tønnessen & Johnsen, 1982), the falsification of data lessened, resulting in the observed increase in whale size.

### Largest Mysticete, Largest Cetacean, Largest Mammal, Largest Metazoan: Blue Whale, *Balaenoptera musculus* (Linnaeus, 1758)

*Balaenoptera musculus* is the largest metazoan to ever exist on Earth. With a global distribution, three widely recognized subspecies occur: *B. m. musculus* in the North Atlantic and North Pacific, *B. m. intermedia* in the Southern Ocean, and *B. m. brevicauda* in the Indian Ocean and South Pacific Ocean (Rice, 1998). The longest individual in our dataset was 33.0 m and caught on May 3, 1930 in Antarctic waters (62.47°S, 32.77°E). A total of 88 individuals in the dataset longer than 30 m were caught between 1916 and 1949. All but one of these were caught in the Southern Ocean. Guinness Records places the largest individual as a female landed in 1909 at South Georgia Island at a length of 33.58 m (Carwardine, 1995). However, there is some uncertainty about how the lengths were recorded in earlier years (before the 1920s), as outlined by Branch et al. (2007). The official method of measuring was from the tip of the snout to the notch between the tails, since the tails were usually cut off after being killed (to prevent currents shifting the bodies before they could be brought to the processing ships). In addition, some early measurements may have been made in Norwegian feet (0.314 m) instead of British feet (0.3048 m), and there is additional estimation error during years when blue whales were processed alongside vessels. For these reasons, early length measurements in excess of 30.5 m should be treated with suspicion. Data for the mass of a complete individual of *B. musculus* do not exist. Mass estimates were derived by whalers by adding up the known capacity of cookers filled with sectioned whales plus estimates for the lost blood and other body fluids (Carwardine, 1995). The two largest estimates are 190 and 199 metric tons.

The large size of *B. musculus* may be linked to its distribution and concentrations of its prey. In response to dense but sparsely distributed patches of krill, blue whales likely evolved great size in order to move efficiently from one feeding ground to the next (Croll et al., 1998). To make their long migratory journeys in response to changing seasons and productivity, whales store energy in the form of thick blubber, so that larger size confers greater starvation resistance (Lockyer, 1981). Indeed, it has been suggested that the observed differences in size between Northern Hemisphere and Southern Hemisphere blue whales is the result of the longer amount of time that Southern blue whales spend away from their feeding grounds (Brodie, 1975).

However, there are limitations to this maximum size. *Balaenoptera musculus* expends a tremendous amount of energy while feeding; consequently, the high costs of lunge-feeding have been found to constrain blue whale distribution to areas with dense prey aggregations (Acevedo-Gutierrez, Croll & Tershy, 2002), and prey distribution therefore likely ultimately constrains the ultimate size of *B. musculus*.

Size data for *B. musculus* was obtained from the International Whaling Commission’s whaling records (data held by IWC). Extending as far back as the 1880s, the records detail the ocean basin where individuals were caught, the specific geographical coordinates of capture, the date of capture, and the sex of each individual. These detailed individual data are available for 84% of all recorded blue whale catches (Branch et al., 2007). Prior to the 1880s, blue whales were too fast to be tracked down by sail-powered vessels, and the technology was insufficient to prevent their carcasses from sinking after death. The total length of individuals was measured from whales at rest on a flat surface, from the notch of the tail fluke to the tip of the upper jaw. The overall distribution of total lengths for *B. musculus* was found to be left-skewed (Quantiles: 75% 25.3 m, 90% 26.5 m, and 95%: 27.1 m, Table 3, Fig. 37). Since these are capture records, the left-skew of the distribution reflects several factors: targeting of the largest individuals, the cessation of physical growth after adolescence, and regulations which forbade capturing blue whales shorter than 21.3 m. It is unclear the extent that this distribution reflects the natural size distribution of *B. musculus* or simply the whaling preference for larger individuals. Sizes also differed between sexes, with females being larger than males (*p* < 2 × 10^−16^, Fig. 38). Considerable geographic variation exists in total length (Fig. 38). The variation seen in the size of *B. musculus* across the different oceans is consistent with the subspecies currently identified. Antarctic blue whales (*B. m. intermedia*) are known to be the largest subspecies, and were longer on average than other locations. At the 95th percentile, both land-based and pelagic whaling catches of *B. m. intermedia* were greater than 27.1 m long. In contrast, the Indian Ocean catches were much smaller, consistent with their designation as *B. m. brevicauda*, or pygmy blue whales, which have a maximum length of 24.1 m (Branch et al., 2007). The North Atlantic and North Pacific blue whales were an intermediate length between *B. m. brevicauda* and *B. m. intermedia*, and are designated as *B. m. musculus*. South-east Pacific blue whales are currently designated as *B. m. brevicauda*, and are an intermediate length between *B. m musculus* and *B. m. intermedia*. As Branch et al. (2007) argued, the lengths of the South-east Pacific blue whales along with their geographic isolation and genetic differences from other blue whale populations, Torres-Florez et al. (2014) suggest that they are a separate population and perhaps a new subspecies that remains unnamed.

The causes of the evolution of the various subspecies have not been given much attention. They are known to have distinct geographic ranges and varying migration patterns, suggesting that these factors have contributed towards the size differences seen today (Gilpatrick & Perryman, 2008). Size differences may result from differences in food availability, as it is well known that the Southern Ocean is a particularly productive system (Tynan, 1998), which could have allowed *B. m. intermedia* to attain larger sizes than other subspecies. More research is needed in order to conclusively determine the cause of the variation in body size seen in the subspecies.

The temporal patterns in blue whale body sizes (Fig. 39) are explained by political situations: the development of better technology to hunt and process whales, the discovery of a large Antarctic population of *B. musculus* that led to a switch from the older hunting sites to the Antarctic in the early 1900s, the occurrences of the World Wars, and changes in policy (Tønnessen & Johnsen, 1982). Overall, the combined size data across all basins demonstrates a significant reduction in length over time (*p* < 2.2 × 10^−16^). This pattern may result from a shift over time from Antarctic to pygmy blue whales, which would result in mean changes in mean length, starting in 1958 when pygmy blue whales were discovered. The decrease in the maximum size of whales caught may also reflect the impact of whaling activities, as well as more rigorous measurement methods. In 1937, minimum catch length was limited to 70 feet (21.4 m) for pelagic caught specimens and 65 feet (19.8 m) for land-based catches (Tønnessen & Johnsen, 1982). The 70 feet limit proved ineffective as *B. musculus* reaches sexual maturity at 77–78 feet (Branch & Mikhalev, 2008); there was frequent “whale stretching” where measurements of shorter whales were reported to be 70 ft (Branch et al., 2007), and changes to policy and new limits were met with strong resistance (Tønnessen & Johnsen, 1982). In 1965 the I.W.C. passed legislation protecting *B. musculus*, which lead to the cessation of whaling data at that time. By 1973, at their low point, the Antarctic populations of *B. musculus* had crashed to just 0.15% of original levels (Branch, Matsuoka & Miyashita, 2004). It is unclear if current size distributions have recovered, as no systematic size data have been collected since the cessation of whaling. Regionally however, in the Northwest Pacific Ocean, blue whales have made a comeback and are at 97% of original levels (Monnahan, Branch & Punt, 2015).

## Conclusions

What are the largest sizes the largest marine megafauna can reach? This is a simple question with a difficult and complex answer. Obtaining the data to address this question was met with several hurdles. As a team we collected and synthesized a considerable amount of literature—newsletters, popular news outlet, books, scientific literature, grey literature, and much more. Additionally, we acquired data from museum samples, online sale and auction sites, and by reaching out to colleagues. Even after this monumental task, however, we often lacked the data necessary to fully explore size variation in these species. First, body size data for some species was completely lacking (e.g., lion’s mane jellyfish and Japanese spider crab), not well explored over the species geographic range (e.g., giant barrel sponge), or considerably outdated (e.g., giant clam). Second, for some species, although data were collected through stranding or nesting networks (e.g., ocean sunfish or leatherback turtle), there was a lack of open collaboration and data sharing between individual networks or with data users. This made a complete assessment of intraspecific size variation over the species ranges difficult to impossible. Third, because of the remoteness and rarity of some of the species (e.g., giant squid and oarfish), much of our data of size was limited to individuals that were dead or dying and washed ashore or stranded in shallow water. Thus, size estimates for these species are not reflective of the healthy population. Fourth, for those organisms inhabiting the deep ocean, adequate sampling and measurement of size is currently technological unfeasible (e.g., giant tube worms). Fifth, the sheer magnitude of some species (e.g., whale sharks and blue whales) makes the quantification of body size extremely challenging. Sixth, and the hardest for us to assess, is how human measurement and collection bias influenced the size patterns found here.

The complexity of answering the question of how large these species can get also depends how we define size. The focus of the popular media and, to a lesser extent, the scientific literature is often the largest individuals of the largest species in the ocean. However, these individuals may reach these extraordinary large sizes through developmental or genetic defects and may not represent the healthiest or, in evolutionary terms, the fittest. For example, Robert Wadlow is considered the tallest person in recorded history at 8 foot and 11 inches (2.72 m), but he needed to wear leg braces to walk and possessed limited tactile sense in his extremities (Drimmer, 1991). At age 21, he passed away from complications due to an infection aggravated by an autoimmune disease. Likewise, the tallest woman, Zeng Jilian, suffered from a severely deformed spine and died at age 17; she reached her great height due to a tumor on her pituitary gland (Tang, 2012). These examples highlight the unreasonable assumption that body size record holders are typical and medically fit. The tallest average size for geographic region in males occurs in the Dinaric Alps of Southern Europe; at 1.86 m this is considerably lower than the 2.72 m of Robert Wadlow. Of course, geographic variation also exists in the average heights of *Homo sapiens*—the average is just 1.58 m in Indonesia (average human heights are available at https://en.wikipedia.org/wiki/Human_height). In this size variation lies true beauty. The largest recorded giant squid is 12 m in length, yet 75% of all individuals ever measured of this species are below 9.2 m and the median is a mere 7.3 m. All of the species here tell a similar size tale, e.g., the maximum reported length for the sperm whale is 24 m but 95% of individuals measured are below 15 m and 75% are below 14.3 m. We must also consider that most species in the ocean, and on Earth, are small (May, 1988; McClain & Boyer, 2009) . By focusing both on the largest species and the largest individuals of them, we concentrated on, ironically, the smallest fraction of life in the oceans.

When possible, we have provided the distribution of sizes within each of the 25 species here (Table 3). This is again to highlight that the maximum reported size is considerably different than the mean and median sizes. This is important both statistically and biologically. Models based on the assumption of maximum size measured as the largest known individual will undoubtedly yield erroneous results. Biologically, there is also importance in distinguishing between what may be the maximum size of a species, limited by anatomical and physiological constraints, and the optimal size, which is the size where an individual would yield the greatest reproductive output, i.e., fitness (Sebens, 1982; Sebens, 1987; Sebens, 2002). Our data here suggest that most individuals operate far below the maximum size set by these ultimate constraints and more likely hover around an optimal size. This optimal size is of course context dependent and based on the environment in which a population finds itself; from this intraspecific size variation arises.

In addition to documenting size variation, we also set out to examine the processes controlling size in the species. We might ask two questions with this regard: 1. Why are these species so large?, and 2. Why are these species not larger? In some cases (e.g., giant squids, giant clams, giant barrel sponges, and walruses) it is clear that mortality decreases with larger size and this relationship likely reflects a decrease in predation pressure with increased size. Larger size also often reflects ecological opportunity in solving energy requirements—large sizes both afford greater caloric intake and reflect the opportunity of greater calories. The giant tube worm circumvents the size constraints of locomotion with a hydrodynamic skeleton, and by actively foraging for food, being sessile and housing symbiotic bacteria that feed off of read supply of hydrothermal fluids. The giant clam relies upon symbiotic photosynthetic algae to provide nutrition, thus reaching sizes much greater than other bivalves. Larger sizes in planktivores like the whale shark and blue whale reflect the size required, in terms of speed and distance, to migrate to high concentration food patches. These larger sizes also may confer starvation resistance when food availability is ephemeral, a hypothesis that may also explain the larger sizes of the giant isopod. Geographic variation in the sizes of these species may also reflect geographic variation in food availability (Sims, Fox & Merret, 1997; Sims et al., 2009; McClain et al., 2012b). Migrations of ocean sunfish may reflect increased productivity in temperate waters in the summer. Larger sizes of bluntnose sixgill sharks in the Northern Hemisphere may be indicative of higher production in these oceans. Likewise, the larger sizes of Blue Whales in the Southern Oceans and smaller sizes in the Indian Ocean likely reflect differences in ocean productivity. Conversely, the constraints on why species are not larger seems to be largely anatomical. Larger giant barrel sponges are at greater risk of dislodgement; the size of giant pacific octopus is limited by a blind gut; Japanese spider crab an exoskeleton; and whale sharks a cartilanguous skeleton.

John Steinbeck in *The Log from the Sea of Cortez* noted,

*“There is some quality in man which makes him people the ocean with monsters and one wonders whether they are there or not. In one sense they are, for we continue to see them…Men really do need sea-monsters in their personal oceans.”*

Indeed, the ocean is populated with monsters or, as we prefer, ocean giants. giant barrel sponges with 2.5 m diameters, Nomura’s jellyfish weighing 200 kg, Japanese spider crabs with 3.7 m leg spans, Australian trumpet snails with shells 0.722 m long, 18.8 m long whale sharks, 7.13 m long great whites, 8 m long oarfish, walruses that weigh 1,883 kg, and of course blue whales that can reach lengths of more than 30 m all inhabit the oceans. The Victorian era mathematician Augustus De Morgan expanded on this with a similar verse

“*Great fleas have little fleas upon their backs to bite ’em,*

*And little fleas have lesser fleas, and so ad infinitum.*

*And the great fleas themselves, in turn, have greater fleas to go on,*

*While these again have greater still, and greater still, and so on*.”

## Additional Information and Declarations

### Data Deposition

The following information was supplied regarding the deposition of related data: Dryad: http://dx.doi.org/10.5061/dryad.411mv.
